# Aspartic proteases modulate programmed cell death and secondary cell wall synthesis during wood formation in poplar

**DOI:** 10.1093/jxb/erac347

**Published:** 2022-08-30

**Authors:** Shenquan Cao, Mengjie Guo, Jiyao Cheng, Hao Cheng, Xiaomeng Liu, Huanhuan Ji, Guanjun Liu, Yuxiang Cheng, Chuanping Yang

**Affiliations:** State Key Laboratory of Tree Genetics and Breeding, Northeast Forestry University, Harbin 150040, China; State Key Laboratory of Tree Genetics and Breeding, Northeast Forestry University, Harbin 150040, China; State Key Laboratory of Tree Genetics and Breeding, Northeast Forestry University, Harbin 150040, China; State Key Laboratory of Tree Genetics and Breeding, Northeast Forestry University, Harbin 150040, China; State Key Laboratory of Tree Genetics and Breeding, Northeast Forestry University, Harbin 150040, China; State Key Laboratory of Tree Genetics and Breeding, Northeast Forestry University, Harbin 150040, China; State Key Laboratory of Tree Genetics and Breeding, Northeast Forestry University, Harbin 150040, China; State Key Laboratory of Tree Genetics and Breeding, Northeast Forestry University, Harbin 150040, China; State Key Laboratory of Tree Genetics and Breeding, Northeast Forestry University, Harbin 150040, China; University of Manchester, UK

**Keywords:** Aspartic protease, *Populus trichocarpa*, programmed cell death, saccharification, secondary cell wall, wood formation, xylem fibre

## Abstract

Programmed cell death (PCD) is essential for wood development in trees. However, the determination of crucial factors involved in xylem PCD of wood development is still lacking. Here, two *Populus trichocarpa* typical aspartic protease (AP) genes, *AP17* and *AP45*, modulate xylem maturation, especially fibre PCD, during wood formation. *AP17* and *AP45* were dominantly expressed in the fibres of secondary xylem, as suggested by GUS expression in *APpro::GUS* transgenic plants. Cas9/gRNA-induced *AP17* or *AP45* mutants delayed secondary xylem fibre PCD, and *ap17ap45* double mutants showed more serious defects. Conversely, *AP17* overexpression caused premature PCD in secondary xylem fibres, indicating a positive modulation in wood fibre PCD. Loss of *AP17* and *AP45* did not alter wood fibre wall thickness, whereas the *ap17ap45* mutants showed a low lignin content in wood. However, *AP17* overexpression led to a significant decrease in wood fibre wall thickness and lignin content, revealing the involvement in secondary cell wall synthesis during wood formation. In addition, the *ap17ap45* mutant and *AP17* overexpression plants resulted in a significant increase in saccharification yield in wood. Overall, *AP17* and *AP45* are crucial modulators in xylem maturation during wood development, providing potential candidate genes for engineering lignocellulosic wood for biofuel utilization.

## Teaser

*Populus*typical aspartic protease genes *AP17*and *AP4*5 function in xylem maturation during wood formation, providing potential candidate genes for engineering lignocellulosic wood for biofuel utilization.

## Introduction

Wood, a dominant terrestrial biomass, is an important renewable resource for construction, paper manufacturing and bioenergy, serving as an essential sink for carbon deposition, recycling atmospheric CO_2_ to preserve the ecological environment. In angiosperm trees, wood is mainly composed of secondary xylem tracheary elements (TEs) and fibres responsible for water transport and mechanical support. Wood formation undergoes a genetically controlled xylogenesis process of xylem development, including cell division of the vascular cambium, cell differentiation and expansion, secondary cell wall (SCW) lignification and deposition, and programmed cell death (PCD) (Mellerowicz and Sundberg, 2008; Déjardin *et al.*, 2010). To date, most biosynthesis enzymes for wood components (cellulose, xylan, and lignin) have been functionally characterized, and significant progress has provided insights into vascular cambium activity, xylem cell differentiation and expansion, and SCW synthesis and regulation (Baucher *et al.*, 2007; Scheller and Ulvskov, 2010; McFarlane *et al.*, 2014; Zhang *et al.*, 2014a; Barros *et al.*, 2015; Zhong *et al.*, 2019). However, only a few studies have documented xylem PCD during wood formation. Thus, the crucial factors involved in tree stem xylem maturation remain to be identified.

In plants, PCD occurs as an inherent part of development (developmental PCD, dPCD) and in responses to environmental stimuli (environmental PCD, ePCD) (Coll *et al.*, 2011; Daneva *et al.*, 2016; Huysmans *et al.*, 2017; Kabbage *et al.*, 2017). A variety of dPCD events in plants have been distinguished based on their developmental context. Differentiation-induced dPCD serves as an inherent differentiation step in particular cell types, such as xylem and root cap cells (Escamez and Tuominen, 2014; Kumpf and Nowack, 2015). In angiosperms, the xylem develops an interconnected network consisting of TEs, fibres, and ray parenchyma cells. In the process, xylem PCD is an inseparable part of xylem maturation, and there is difficulty uncoupling cell death from SCW formation. Owing to a breakthrough in the *Zinnia elegans* TE differentiation system *in vitro* (Fukuda and Komamine, 1980), more information on the PCD of TEs has been provided in angiosperm species. A striking feature is the rupture of the tonoplast, which releases hydrolytic enzymes from the vacuole, and activation of cytoplasmic enzymes by acidification of the cytoplasm, rapidly initiating post-mortem clearance, dismantling the membrane system, and degrading nuclear DNA and organelles (Fukuda, 1997; Groover *et al.*, 1997; Kuriyama, 1999; Obara *et al.*, 2001; Escamez and Tuominen, 2014). Based on morphological changes during xylem cell death, the main differences in PCD of fibres are that, apart from vacuolar disintegration, nuclear DNA integrity is compromised, and cytoplasmic contents are gradually degraded (Courtois-Moreau *et al.*, 2009; Bollhöner *et al.*, 2012). Overall, genetic evidence for understanding the dPCD pathway is still lacking, especially for xylem PCD during wood formation.

Proteases, which are crucial regulators, have been implicated in the regulation and/or progression of PCD processes in plants (Minina *et al.*, 2014; Buono *et al.*, 2019). Arabidopsis xylem cysteine proteases XCP1 and XCP2 accumulate in the vacuole; moreover, deletion of the encoding genes does not cause PCD impairment but shows a delay in PCD-associated cell clearance during TE differentiation (Funk *et al.*, 2002; Avci *et al.*, 2008). Han *et al.* (2012) indicated that the 20S proteasome with caspase-3-like activity in poplar xylem is involved in PCD during TE differentiation. AtMC9, a xylem-specific metacaspase located in the apoplast and vacuole, participates in the regulation of TE autolysis in Arabidopsis, and the *atmc9* mutant displays delayed post-mortem clearance (Vercammen *et al.*, 2006; Bollhӧner *et al.*, 2013; Escamez *et al.*, 2016). The MC9 poplar homologues, PttMC13 and PttMC14, modulate the downstream proteolytic processes and cell death of xylem elements (Bollhӧner *et al.*, 2018). Mutations in the Arabidopsis cysteine protease gene *CEP1* cause delayed organelle degradation in fibres and TEs during xylem development (Han *et al.*, 2019). However, fewer xylem proteases have been functionally described in xylem PCD of wood formation at the genetic level.

In our previous study, the characterization of aspartic protease (AP) genes in *Populus trichocarpa* suggested that multiple APs might participate in wood formation (Cao *et al.*, 2019). In plants, APs include typical, atypical, and nucellin-like APs, and several atypical and nucellin-like APs have been implicated in dPCD processes and stress responses (Ge *et al.*, 2005; Phan *et al.*, 2011; Niu *et al.*, 2013; Soares *et al.*, 2019). Typical APs have a plant-specific insert (PSI) domain as a vacuolar sorting signal (Pereira *et al.*, 2013). In this study, two typical AP genes, *AP17* and *AP45*, are predominantly expressed in the fibres of the secondary xylem in *P. trichocarpa*. Cas9/gRNA-induced gene mutants and overexpression transgenic plants indicate that *AP17* and *AP45* play a positive role in xylem PCD during wood formation. In addition, loss or overexpression of *AP17* and *AP45* alters wood SCW synthesis, especially by decreasing lignin content. These alterations in wood SCWs result in a significantly improved saccharification yield, applicable to biofuel utilization.

## Materials and methods

### Plant material and growth conditions

#### P.

*trichocar*pa genotype Nisqually-1 grown in a greenhouse at the Northeast Forestry University of China was used in this study. Sterile plantlets were propagated as previously described (Li *et al.*, 2017) for genetic transformation in a growth chamber. The transgenic plantlets generated were planted in soil for phenotypic analysis in a greenhouse (22−25 °C; 16/8h light/dark cycle) with a light intensity of ~250 μmol m^−2^ s^−1^. *Arabidopsis thaliana* (ecotype Col-0) seedlings were used for transformation, and the transgenic Arabidopsis plants obtained were grown for phenotypic analysis under long-day conditions (20−22 °C; 16/8h light/dark cycle; 80−120 μmol m^−2^ s^−1^ light intensity).

### Vector constructs

Genomic DNA from *P. trichocarpa* was extracted using a plant genomic DNA extraction kit (Bioteke, China). Promoter regions of approximately 3 kb of *AP6/11/19/42/45/47* were amplified from genomic DNA with specific primers, and the purified fragments were ligated into pENTR/D-TOPO (Invitrogen, USA) for sequencing. These clones were constructed into pGWB3 vectors using the LR (*att*L1-gene-*att*L2 × *att*R1-ccdB-*att*R2) reaction mediated by Gateway^®^ LR Clonase™ (Invitrogen, USA). The *AP17* promoter fragment was constructed into pGWB3 vector using the LR reaction, which was obtained from a previous study (Cao *et al.*, 2019).

Efficient gRNA target sites for *AP17* and/or *AP45* were analysed using CRISPR direct software for designing CRISPR/Cas gRNA with reduced off-target sites (Naito *et al.*, 2015). The PCR fragment was amplified using pCBC-DT1T2 as a template, and the purified PCR fragments and pHSE401 plasmid were generated using pHSE401-2gRNA vectors with the Golden Gate reaction. pCBC-DT1T2 and pHSE401 were kindly provided by Prof. Qi-Jun Chen (Xing *et al.*, 2014).

For the overexpression constructs, the coding DNA sequence (CDS) of the *AP17*/*45* gene was amplified and ligated into pGWB11 to generate the *35S::AP17/45* construct. Overlapping PCR was used to splice the promoter and the CDS of the *AP17* gene, and the splicing fragment was integrated into pGWB10 to generate the *proAP17::AP17* construct. The mutated forms of *AP17*^*D106/293N*^ and *AP45*^*D106/293N*^ were constructed by site-directed mutagenesis using the TaKaRa MutanBEST Kit (TaKaRa, China), and the resultant clones were integrated into pGWB10 or pGWB11 to generate *35S::AP17*^*D106/293N*^, *35S::AP45*^*D106/293N*^, and *proAP17::AP17*^*D106/293N*^ constructs. The primers used are shown in Supplementary Table S1. The resultant vectors were introduced into the *Agrobacterium tumefaciens* strain GV3101 for transformation.

### Genetic transformation of *P. trichocarpa* and *A. thaliana*

*Agrobacterium*-mediated transformation of *P. trichocarpa* was performed according to the protocol described by Li *et al.* (2017) and Xu *et al.* (2021). *A. thaliana* was transformed using the floral-dip method as described previously (Clough and Bent, 1998). Transgenic Arabidopsis plants were selected for kanamycin resistance and assayed using PCR.

### Identification of the Cas9/gRNA-induced mutations

After 1 month of growth of the transformants in a greenhouse, genomic DNA was extracted from the leaves of WT and transgenic plants and used as the template for PCR amplification with primers (Supplementary Table S1) flanking the gRNA target sites. The amplified DNA fragments were cloned into pMD18-T vectors (TaKaRa), and 30 positive clones from each amplicon were sequenced to detect the mutations as described previously (Xu *et al.*, 2021).

### GUS staining

GUS staining analysis of transgenic young trees was performed as previously described (Jefferson *et al.*, 1987). For each *promoter::GUS*, at least six independent transgenic lines were used for GUS staining analysis, and the experiments were repeated thrice. Images of the stained leaves were captured using a two-colour infrared laser imaging system (Odyssey, USA); and images of the roots, stems, and veins were captured using a BX43 upright microscope (Olympus, Japan); and images of apical buds were recorded using a SZX7 stereomicroscope (Olympus, Japan).

### Cell viability and death assays by nitroblue tetrazolium, Evans blue, Coomassie blue, and trypan blue staining

Cell viability and death analysis were performed as described previously (Escamez *et al.*, 2017). Cross-sections of the 22^nd^ stem internodes from 4-month-old wild-type (WT) and transgenic young trees were collected by hand-sectioning. After the cross-sections were incubated for 2 h under strong light in 0.1 M phosphate buffer (pH 7.6) containing 1 g l^−1^ nitroblue tetrazolium (NBT) (Sigma-Aldrich) and 0.1 M sodium succinate, the width of living xylem (four random positions per section and three sections per tree) was measured under a BX43 upright microscope. For Evans blue staining, 70-cm-long *Populus* stems were incubated in 1% Evans blue solution for 2 h in the greenhouse, then cross-sections of 15^th^ stem internodes were collected by hand-sectioning, and recorded under a BX43 upright microscope.

Coomassie staining was carried out according to Brereton (2016) with minor modifications. Transverse sections of 60 μm were made on a vibratome (Leica, Germany) and immediately incubated in 0.1 M phosphate buffer (pH 7.6) containing 1% Coomassie blue R250 and 0.1 M sodium succinate for 4 h under room temperature. After washing with the corresponding phosphate buffer, the sections were visualized under a BX43 upright microscope. Cross-sections of basal stems of Arabidopsis plants (growth stage 9.70; Boyes *et al.*, 2001) were collected by hand-sectioning and incubated in NBT solution as above for 5 min. The cross-sections were viewed under a BX43 upright microscope.

Trypan blue staining to visualize cell death was performed as previously described (Wang *et al.*, 2019). Briefly, the 4^th^ leaves from 3-week-old transgenic Arabidopsis plants (growth stage 1.10) were harvested and immersed for 20 min in reaction buffer (65 °C) containing 2 mg/ml trypan blue (Sigma-Aldrich), 25% (w/v) lactic acid, 23% (v/v) liquid phenol, and 25% (v/v) glycerol. These stained leaves were then destained with 250% (w/v) chloral hydrate solution and recorded using a SZX7 stereomicroscope. Twelve individual plants for each transgenic line were prepared, and three independent experiments were repeated.

### Transmission electron microscopy (TEM) analysis

Samples were prepared for transmission electron microscopy (TEM) analysis according to the method described previously (Xi *et al.*, 2017). The 12^th^ stem internodes of 3-month-old *Populus* young trees were cut into lengths of 2–3 mm and fixed in 0.1 M PBS buffer (pH 7.4) containing 2.5% (w/v) glutaraldehyde and 4% (w/v) paraformaldehyde (Sigma-Aldrich). The samples were sequentially washed, dehydrated, immersed, sectioned, and stained, and the images were recorded using an HT-7700 electron microscope (Hitachi, Japan) at 80 kV. Three sections per tree and 15 measurements per section were recorded using Image J software. In addition, cross-sections of basal stems of Arabidopsis plants (growth stage 6.90) were collected by hand-sectioning and then stained with 0.05% (w/v) toluidine blue solution for 5 min. Images of the cross-sections were captured using a BX43 upright microscope, and the wall thickness of the interfascicular fibres (IFs) was measured using Image J software.

### Wood fibre and vessel cell size analyses

The sizes of the wood fibre and vessel cells were determined as described previously (Lautner *et al.*, 2007) with minor modifications. The 22^nd^ stem internodes of 4-month-old WT and transgenic trees were peeled, cut into small pieces, and macerated in a solution containing 10% (v/v) nitric acid and 10% (v/v) chromic acid for 4–6 h at 60 °C. Each stem internode was then rinsed lightly with distilled water six times. After rinsing and mechanical breakage, secondary xylem cells were placed on a slide and visualized with 0.1% (w/v) fuchsin acid under a BX43 upright microscope. The sizes of 900 fibres and 600 vessels from each sample were measured using ImageJ software.

### Wood composition assay

The basal stems from 4-month-old *P. trichocarpa* young trees were peeled and dried at 55 °C, and the dried wood was ground to a fine powder by ball milling. The powder was washed successively with 70% ethanol, a chloroform/methanol (1:1 v/v) solution, and acetone, and the air-dried insoluble residues were processed into cell wall materials for crystalline cellulose and lignin content assays. The lignin content was assayed by the acetyl bromide spectrophotometric method (Foster *et al.*, 2010a), and the crystalline cellulose content was determined as described previously (Foster *et al.*, 2010b).

### Transcriptome sequencing

Samples of developing xylem were scraped from three 4-month-old *P. trichocarpa* tree stems 25–35 cm above soil level. Total RNA was extracted using a plant RNA extraction reagent (Bio-Flux, China). Three biological repeats of *ap17ap45* and WT RNA preparations were sent to the Annoroad Gene Biotechnology Co., Ltd (Beijing, China) for quality examination, and those that qualified were used for library preparation using the NEBNext® Ultra™ RNA Library Prep Kit (NEB, USA). The libraries were sequenced on an Illumina HiSeq 4000 sequencing platform (San Diego, USA). The raw reads were filtered to generate clean reads and mapped to the *P. trichocarpa* genome using HISAT2 version 2.1.0 (Kim *et al.*, 2015). Read counts for each gene were generated by HTSeq version 0.6.0, and the fragments per kilobase of transcript per million mapped reads (FPKM) were calculated to estimate the expression level of genes in each sample (Trapnell *et al.*, 2012). The data were submitted to the NCBI Sequence Read Archive (accession numbers SRR14040098–SRR14040103).

The transcriptome data processing was performed as previously described (Han *et al.*, 2019). Differential expression analysis between the *ap17ap45* and WT plants was performed using DESeq2 version 1.6.3. The *P*-value was adjusted using the Benjamini and Hochberg’s approach for controlling the false discovery rate (FDR). A corrected *P*-value <0.05 and |log2_ratio| ≥1 were set as the threshold for significant differential expression. Gene ontology (GO) enrichment analysis of differentially expressed genes (DEGs) was performed using the GOseq R package, and a corrected *P*-value of <0.05 was considered significantly enriched among DEGs.

### RT-qPCR

cDNA was synthesized using the PrimeScript RT Reagent Kit (TaKaRa) and diluted 1:10 in nuclease-free water. RT-qPCR was performed on a qTOWER^3^G Real-Time PCR System (Jena, Germany) with the TB Green Premix Ex *Taq* (TaKaRa). The PCR parameters were as follows: 95 °C for 30 s; 40 cycles of 95 °C for 5 s, 55 °C for 30 s, 72 °C for 45s. The comparative Ct (2^−ΔΔCt^ and 2^−ΔCt^) methods were used to calculate transcript abundance in *Populus* and Arabidopsis, respectively. The gene expression levels were normalized using the geometric mean of *Ptactin2*, *PtEF1β*, and *PteIF5A* expression in *Populus*, and *AtActin2*, *AtGAPDH*, and *AtUBQ10* expression in Arabidopsis. The primers are listed in Supplementary Table S1.

### Saccharification assays

Saccharification of the wood cell walls was performed as previously described (Yang *et al.*, 2013). Ball-milled powder from the 4-month-old wood of WT, mutant, and overexpressed plants was prepared. Saccharification assays were initiated by adding 83 mM sodium citrate buffer (pH 6.2) containing 4.4% (w/w) cellulase R10 (Yakult Pharmaceutical Ind. Co., Ltd., Japan) and 0.44% (w/w) β-glucosidase (Yuanye Bio-Technology, China). Reducing sugars were assessed by the 3,5-dinitrosalicylate reaction. After 12, 24, 36, 48, 60, and 72 h of incubation (at 50 °C) in a constant temperature shaking incubator at 200 rpm (Labotery, China), reducing sugars were quantified by measuring the absorbance at 540 nm using a microplate reader (Tecan, Austria).

### Statistical analysis

Data from the transgenic and WT plants were analysed using analysis of variance (ANOVA) in SPSS 19.0. Values are the mean ±SD, and asterisks indicate statistical significance at different levels (*P<0.05, **P<0.01).

## Accession numbers

The sequences from this article can be found in Phytozome (version 13.0) under the following accessions: *AP6* (Potri.001G356900), *AP11* (Potri.002G228300), *AP17* (Potri.004G007600), *AP19* (Potri.005G002800), *AP42* (Potri.010G003400), *AP45* (Potri.011G007600), and *AP47* (Potri.013G002200).

## Results

### Identification of the typical APs dominantly expressed in *P. trichocarpa* secondary xylem

Seven typical APs (*AP6*, *AP11*, *AP17*, *AP19*, *AP42*, *AP45*, and *AP47*) have been shown in *P. trichocarpa* (Cao *et al.*, 2019). We constructed a phylogenetic tree of typical *P. trichocarpa* APs with Arabidopsis, grape and rice homologues (Supplementary Table S2). *AP11*, *AP17*, and *AP45* clustered into a subgroup with Arabidopsis *PASPA3*, which was the closest to *AP11* (Supplementary Fig. S1A). As shown in Supplementary Fig. S1B, the seven typical APs in *P. trichocarpa* have the PSI (including *SapB_1* and *SapB_2*) domain, and most contain a C-terminal vacuolar sorting determinant (FAEA peptide), except for *AP11* and *AP42*. In addition, the conserved aspartic residues in the DTG and DSG motifs, crucial for AP activity (Yao *et al.*, 2012; Li *et al.*, 2016; Guo *et al.*, 2019), are present in all seven typical APs.

**Fig. 1.**
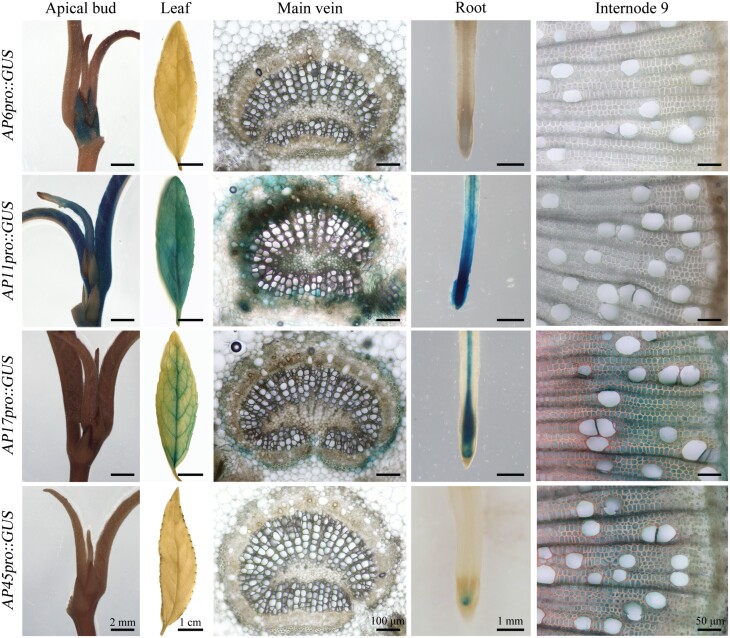
Tissue expression activities of *AP6*, *AP11*, *AP17*, and *AP45* gene promoters in *P. trichocarpa*. GUS activity driven by typical *AP6*, *11*, *17*, or *45* gene promoters was analysed in the corresponding *APpro::GUS* transgenic *P. trichocarpa*. For each *promoter::GUS*, at least six independent transgenic lines were used for GUS staining analysis, and the experiments were repeated thrice with consistent results. Leaves and roots from 4-week-old transgenic plants; apical buds, main veins, and stems from 3-month-old transgenic plants.

To identify the typical APs involved in secondary xylem development, we examined the promoter activities of the seven APs in different tissues through a *promoter::GUS* approach (Fig. 1; Supplementary Fig. S2). Strong GUS signals were detected in apical buds, leaves, and roots of the *AP11pro::GUS* lines. In contrast, *AP17* promoter-driven GUS expression was intensively detected in the fibres of secondary xylem, and leaf veins and root steles, and GUS expression driven by the *AP45* promoter was moderate in secondary xylem fibres. For *AP6pro::GUS* lines, GUS signals were visually localized only in the scales of the leaf bud. In addition, GUS activity was hardly detected in *AP19pro::GUS*, *AP42pro::GUS*, or *AP47pro::GUS* lines grown in a greenhouse under normal conditions (Supplementary Fig. S2). The promoter-driven GUS expression patterns of these typical APs in different tissues were consistent with our previous RT-qPCR and microarray data (Supplementary Fig. S3; Cao *et al.*, 2019). Taken together, *AP17* and *AP45* are dominantly expressed in the fibres of *P. trichocarpa* secondary xylem, suggesting that they might be involved in wood formation.

### Production of Cas9/gRNA-induced *ap17*, *ap45*, and *ap17ap45* mutants

**Fig. 2.**
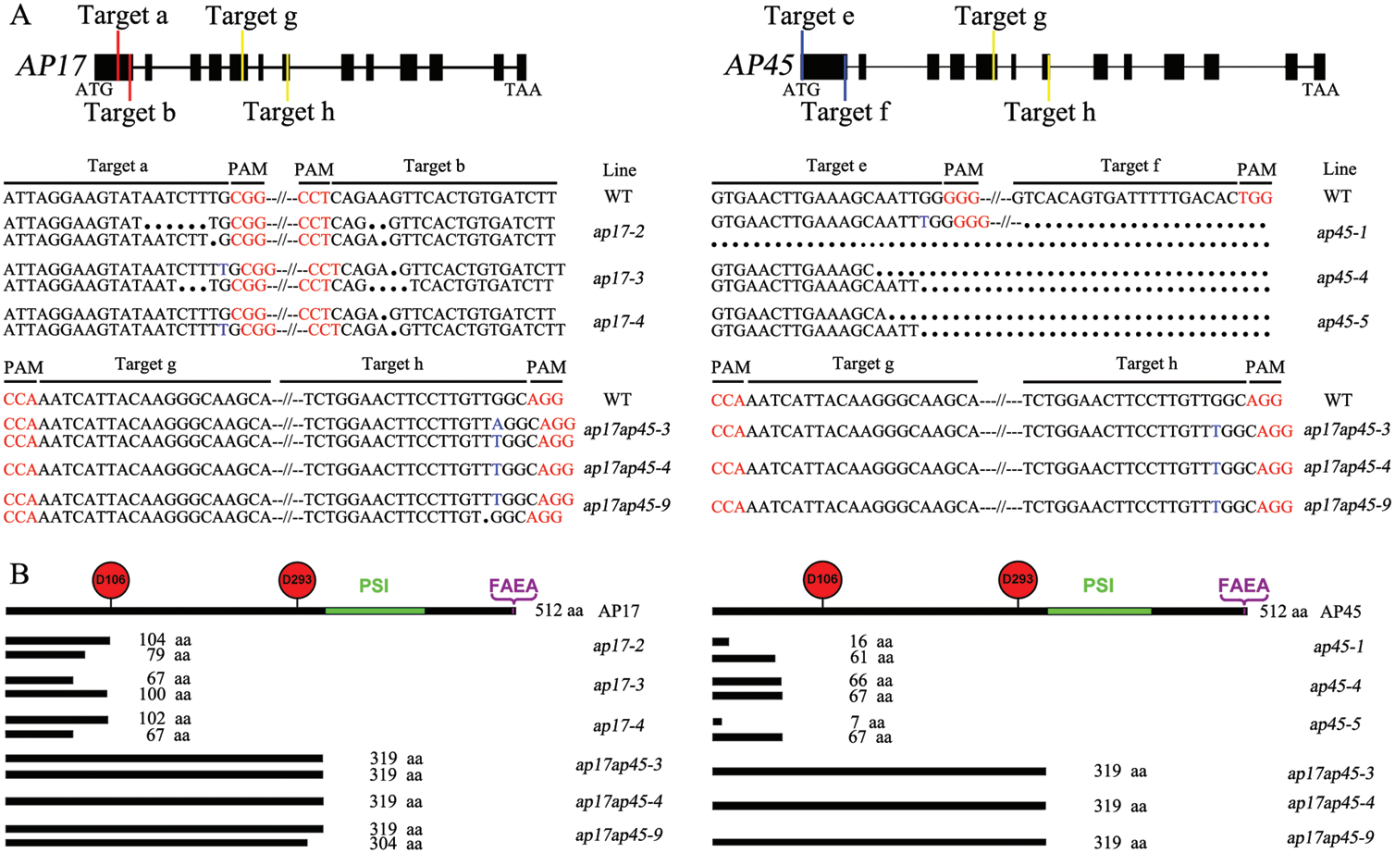
Cas9/gRNA-induced mutations in *AP17*, *AP45*, and *AP17/45* genes. (A) Six gRNAs were designed in the *AP17*, *AP45*, and *AP17/45* genes. Characterization of the target site mutations in nine *ap* mutants (*ap17-2#*, *-3#* and *-4#*, *ap45-1#*, *-4#* and *-5#*, *ap17ap45-3#*, *-4#*, and *-9#*). Black points and blue letters represent the deletion and insertion of nucleotides. (B) The deduced amino acids of protein-coding regions from the Cas9/gRNA-edited genes in the *ap17*, *ap45*, and *ap17ap45* mutants. The number of amino acids of mutated proteins is shown and the conserved catalytic residues, PSI domains and FAEA peptides are indicated in AP17 and AP45.

To investigate the function of *AP17* and *AP45* in *P. trichocarpa*, we generated two gene mutants using the Cas9/gRNA technique. As *AP17* and *AP45* are a pair of duplicated genes and share high amino acid identities (90.2%), double gene mutation lines were also generated using the same method. Two pairs of gRNAs were selected for each gene to generate multiple mutants (Fig. 2A). After detection of the edited target sites, a total of 12, 7, and 10 mutation lines were obtained for the *AP17*, *AP45*, and *AP17/45* genes, respectively (Supplementary Table S3). As a result, the nucleotide deletions and insertions at target sites caused frameshift mutations and generated putative knockouts of these genes. We chose three *ap17* (*ap17-2*, *ap17-3*, *ap17-4*), *ap45* (*ap45-1*, *ap45-4*, *ap45-5*) and *ap17ap45* (*ap17ap45-3*, *ap17ap45-4*, *ap17ap45-9*) knockout mutants for further functional studies (Fig. 2B).

**Fig. 3.**
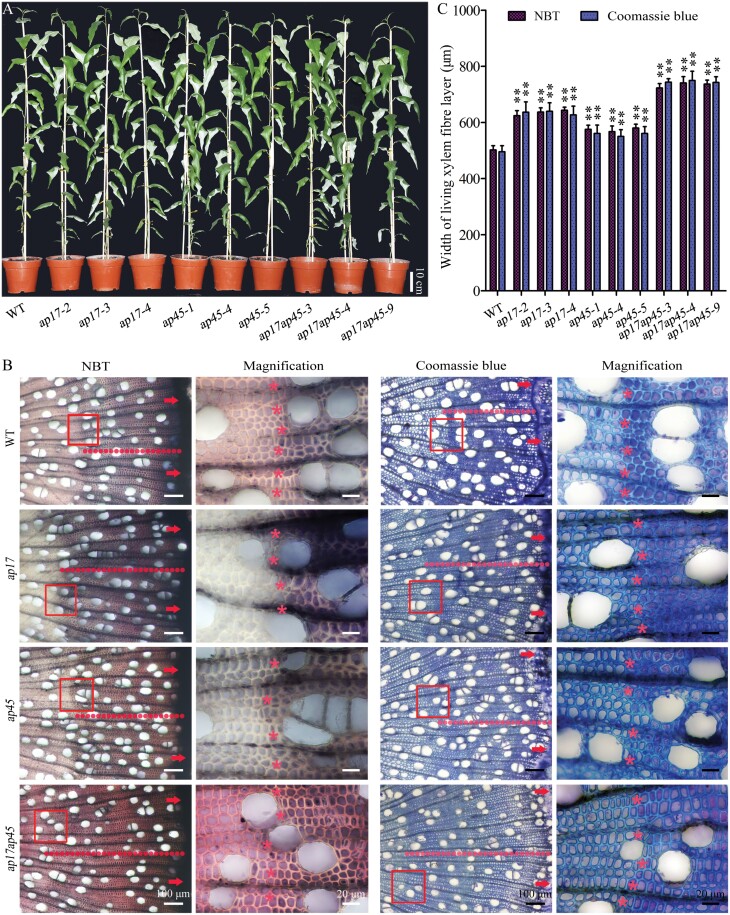
Histochemical staining of living xylem elements with nitroblue tetrazolium (NBT) and Coomassie blue in the WT, and the *ap17*, *ap45*, and *ap17ap45* mutant stems. (A) Morphology of WT, and the *ap17*, *ap45*, and *ap17ap45* mutants grown for 4 months in the greenhouse. (B) NBT and Coomassie staining shows xylem cell viability in transverse sections of 4-month-old WT, and *ap17*, *ap45*, and *ap17ap45* mutant stems. Living xylem cells are black/purple or the remnants of cell contents are blue in the images, and the fibre cells that lose their viabilities or contents were visible synchronously around the circumference of the stems, as partly indicated in red rectangles. After magnification of the red rectangles, asterisks indicate the living xylem fibre cells with black/purple coloration or the remnants of cell contents with blue coloration. Arrowheads indicate vascular cambium and red dotted lines indicate the width of living xylem fibre layer. (C) The width of the living xylem fibre layer is assayed in different lines of the *ap17*, *ap45*, and *ap17ap45* mutants. Values are means ±SD (n=3) from three biological repeats. Asterisks denote significant difference from the WT by one-way ANOVA (**P<0.01).

The WT, and the *ap17*, *ap45*, and *ap17ap45* mutants were grown for 4 months in a greenhouse under the same environmental conditions. Compared with the WT, no significant differences in the phenotypes, including tree height, stem diameter, and leaf shape, were observed in the *ap17*, *ap45*, and *ap17ap45* mutant trees (Fig. 3A; Supplementary Fig. S4A, B). In addition, we measured the sizes of wood fibres and vessels in the WT, and the *ap17*, *ap45*, and *ap17ap45* mutants, and no significant differences were detected between the WT and these mutants (Supplementary Fig. S4C–F). These data suggest that *AP17* and *AP45* have no (or weak) roles in xylem cell proliferation and expansion in *P. trichocarpa*.

### Delay of xylem PCD in wood formation by *ap17*, *ap45*, and *ap17ap45* mutants

To examine whether *AP17* and *AP45* are implicated in PCD of wood formation, we detected cell viability and death using several histochemical staining methods in the xylem of the WT, and the *ap17*, *ap45*, and *ap17ap45* mutants. Cell viability assay with NBT staining showed that the death of xylem fibres occurred synchronously around the circumference of the 4-month-old WT stems at a distance of approximately 520 μm from the vascular cambium (Fig. 3B, on the left). Compared with the WT, the width of the living xylem fibre layer in the *ap17-2* and *ap45-1* lines (from vascular cambium to pith) was increased by 24.3% and 13.0%, respectively, and similar results were observed in other lines of the mutants (Fig. 3C). In addition, the width of the living xylem fibre layer of *ap17ap45* double mutants increased more significantly than the *ap17* or *ap45* single mutants, revealing the redundant roles of *AP17* and *AP45* in xylem fibre cell death. Furthermore, Coomassie staining, that assesses remnants of cell contents, was performed in xylem elements of the WT, and the *ap17*, *ap45*, and *ap17ap45* mutants. Few remnants of cell content in xylem fibres were observed around the circumference of the WT stems at a distance of approximately 510 μm from the vascular cambium (Fig. 3B, on the right), suggesting that these fibres have undergone cellular clearance of PCD. In comparison to the WT, the width of the cell content-enriched xylem fibre layer was significantly increased in the *ap17*, *ap45*, and *ap17ap45* mutants (Fig. 3B, C). These results show that the *ap17* and *ap45* mutants delay fibre cell death during wood formation. Next, xylem vessel cell death in the WT, and the *ap17*, *ap45*, and *ap17ap45* mutants was detected by their ability to transport water using Evans blue staining (Escamez *et al.*, 2017). However, we did not observe a significant delay in xylem vessel cell death in the *ap17* and *ap45* mutants (Supplementary Fig. S5). Taken together, our findings indicate that the loss of *AP17* and/or *AP45* delays xylem maturation during wood formation.

Transcriptome analysis of misregulated genes in developing xylem was performed in the *ap17ap45* mutants, and a total of 126 DEGs were identified in comparison with the WT (Supplementary Table S4). As expected, *AP17* and *AP45* appeared in the downregulated DEG group, and the expression profiles of partial DEGs were verified by RT-qPCR (Supplementary Fig. S6). GO enrichment indicated that transcription regulator and oxidoreductase GO categories were overrepresented among upregulated DEGs, while transcription regulator and peptidase activity were enriched among downregulated DEGs (Supplementary Fig. S7A). We further searched for DEG expression profiles in WT woody tissues using the ASPWOOD database (Sundell *et al.*, 2017). Most upregulated DEGs (41) showed low expression in the cambium and cell expansion (CA-CE) zones, while the downregulated DEGs (41) were highly expressed in the SCW formation and maturation zones (Supplementary Fig. S7B). These DEGs were mainly associated with hormone signalling, transcription regulation, cellular oxidation, the cell membrane and autophagy, transport, protein degradation and SCW synthesis (Supplementary Fig. S7C). Interestingly, most lignin biosynthetic enzyme genes were decreased in *ap17ap45* mutants at the transcriptional level (Supplementary Table S5). Thus, transcriptome data suggest that *AP17/45* affects PCD and SCW synthesis during wood formation.

### Overexpression of *AP17* leads to premature xylem cell death in wood formation

To further investigate the role of *AP17* in wood formation, *proAP17::AP17* and *35S::AP17* overexpression constructs were introduced into *P. trichocarpa*. We obtained 17 and 22 independent transgenic lines for *proAP17::AP17* and *35S::AP17*, respectively. After detection by RT-qPCR analysis, six highly expressed transgenic lines, *proAP17::AP17-2/5/12* and *35S::AP17-4/17/22* (Supplementary Fig. S8), were used for further functional studies.

**Fig. 4.**
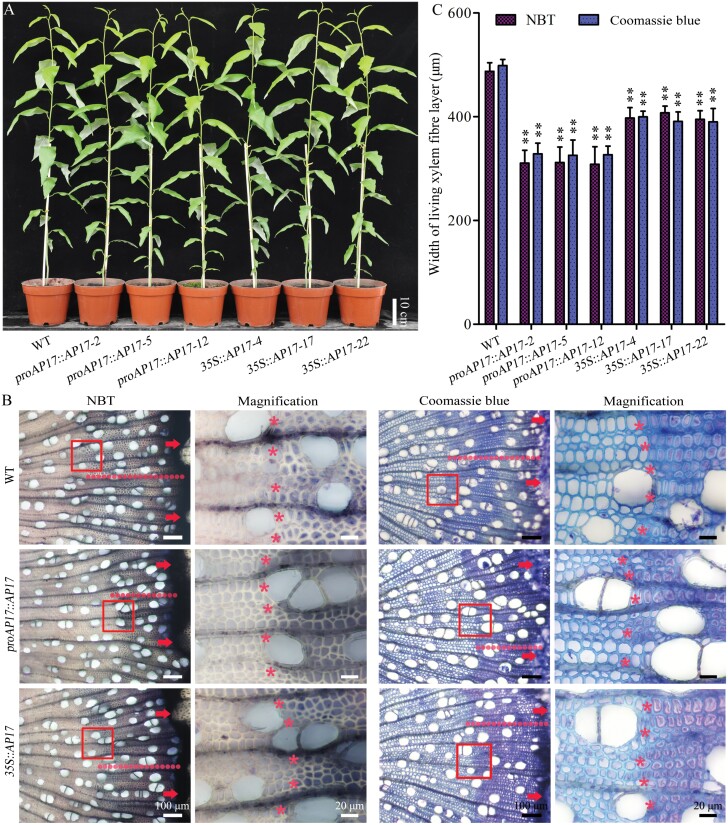
Phenotypes of the *AP17*-overexpressed transgenic trees. (A) Morphology of WT, *proAP17::AP17*, and *35S::AP17* mutants grown for 4 months in a greenhouse. (B) NBT and Coomassie staining shows xylem cell viability in transverse sections of 4-month-old WT, and *proAP17::AP17* and *35S::AP17* transgenic *P. trichocarpa* stems. Living xylem cells are black/purple or the remnants of cell contents are blue in the images, and the fibre cells that lose their viabilities or contents were visible synchronously around the circumference of the stems, as partly indicated in red rectangles. After magnification of the red rectangles, asterisks indicate the living xylem fibre cells with black/purple coloration or the remnants of cell contents with blue coloration. Arrowheads indicate vascular cambium and red dotted lines indicate the width of living xylem fibre layer. (C) The width of living xylem fibre layer is assayed in different *proAP17::AP17* and *35S::AP17* transgenic lines. Values are means ±SD (n=3) from three biological repeats. Asterisks denote significant difference from the WT by one-way ANOVA (**P<0.01).

Compared with the WT, no significant differences in growth phenotypes, including tree height and stem diameter, were observed in *proAP17::AP17-2/5/12* and *35S::AP17-4/17/22* overexpressed plants (Fig. 4A). In addition, the sizes of wood fibres and vessels in these overexpressed plants were the same as those in WT plants (Supplementary Fig. S9). However, xylem cell viability staining showed that the living xylem fibre layer in *proAP17::AP17* and *35S::AP17* overexpression lines was reduced by 35.9–36.6% and 16.3–18.9%, and the overexpression of *AP17* driven by the native promoter accelerated cell death more efficiently than that driven by the *35S* promoter, suggesting that *AP17* overexpression leads to premature fibre cell death (Fig. 4B, C). In addition, Coomassie staining revealed that the width of the cell content-enriched xylem fibre layer in *proAP17::AP17* and *35S::AP17* overexpressing plants was significantly smaller than that in the WT (Fig. 4B, C). However, we did not observe a significant prematurity in xylem vessels of *proAP17::AP17* and *35S::AP17* overexpressing plants (Supplementary Fig. S5). Together with *ap17*, *ap45*, and *ap17ap45* mutant phenotypes (Fig. 3B, C), these findings indicate that *AP17* positively modulates xylem cell death in wood formation.

### Genetic perturbation of *AP17* and *AP45* alters SCW synthesis in wood formation

**Fig. 5.**
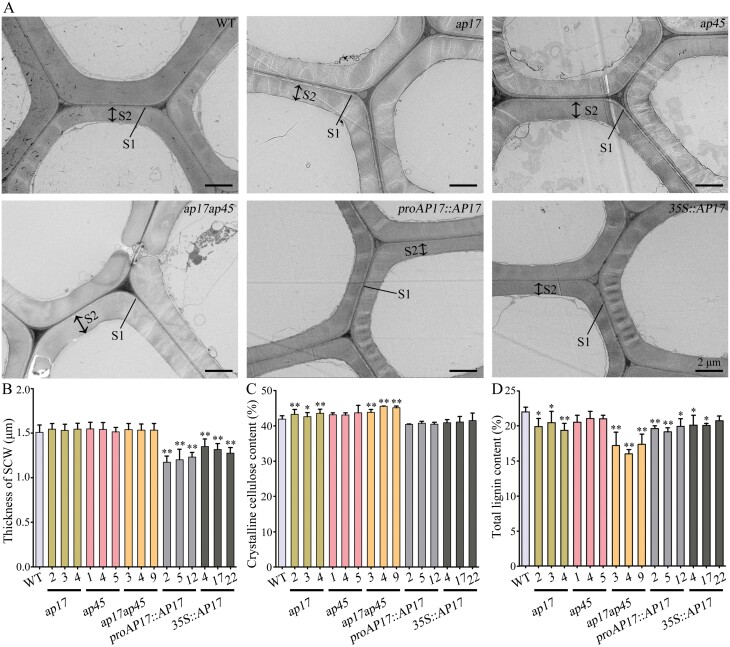
Genetic perturbations of *AP17* and/or *AP45* alter wood SCW synthesis in transgenic trees. (A) TEM images of secondary xylem mature fibre walls in 4-month-old WT, *ap17*, *ap45*, and *ap17ap45* mutants, and *proAP17::AP17* and *35S::AP17* transgenic trees. S1 and S2 layers of fibre SCWs are visible. (B) Wall thickness of secondary xylem mature fibres in 12 stem internodes of 3-month-old WT and transgenic trees. Values are means ±SD (n=15). (C-D) Cellulose and total lignin in dry woods of WT and transgenic trees. Values are means ±SD (n=3) from three biological repeats. Asterisks denote significant differences from the WT by one-way ANOVA (*P<0.05; **P<0.01).

To assess whether *AP17* and *AP45* play a role in SCW synthesis, we measured the wall thickness of xylem fibres in the WT, the *ap17*, *ap45* and *ap17ap45* mutants and the *AP17* overexpression lines. TEM analysis clearly showed a multi-layered wall structure in WT wood fibres with visible S1 and S2 layers and in *ap17*, *ap45*, and *ap17ap45* mutants, mature fibres exhibiting similar wall structures in the wood (Fig. 5A). However, the wall thickness of the developing (immature) fibres in *ap17ap45* mutants was reduced by 3.7–23.2%, compared to WT plants (Supplementary Fig. S10). The *AP17*-overexpressed plants had a similar wood fibre wall structure but a significant reduction in the wall thickness of fibres compared to the WT and mutants (Fig. 5A, B). Compared with the WT, the wall thickness of secondary xylem mature fibres in *proAP17::AP17* and *35S::AP17* overexpression lines was reduced by 18.3–22.2% and 10.6–15.6%, as suggested by the statistical data (Fig. 5B). These results indicate that overexpression of *AP17* abates fibre wall thickening during wood formation.

We further examined the cellulose and lignin content in the wood of *ap17*, *ap45*, and *ap17ap45* mutants, and *AP17* overexpression lines. Compared with the WT, the cellulose content was increased in *ap17* and *ap17ap45* mutants (Fig. 5C). However, the lignin content decreased by 8.4–11.9% and 21.0–27.2% in the *ap17* and *ap17ap45* mutants, respectively (Fig. 5D). Likewise, the lignin content was decreased in *proAP17::AP17* and *35S::AP17* overexpression lines (Fig. 5D). Thus, these findings reveal that genetic perturbation of *AP17* and *AP45* alters SCW synthesis during wood formation.

### Ectopic overexpression of *AP17* or *AP45* accelerates PCD and reduces SCW thickness in Arabidopsis

**Fig. 6.**
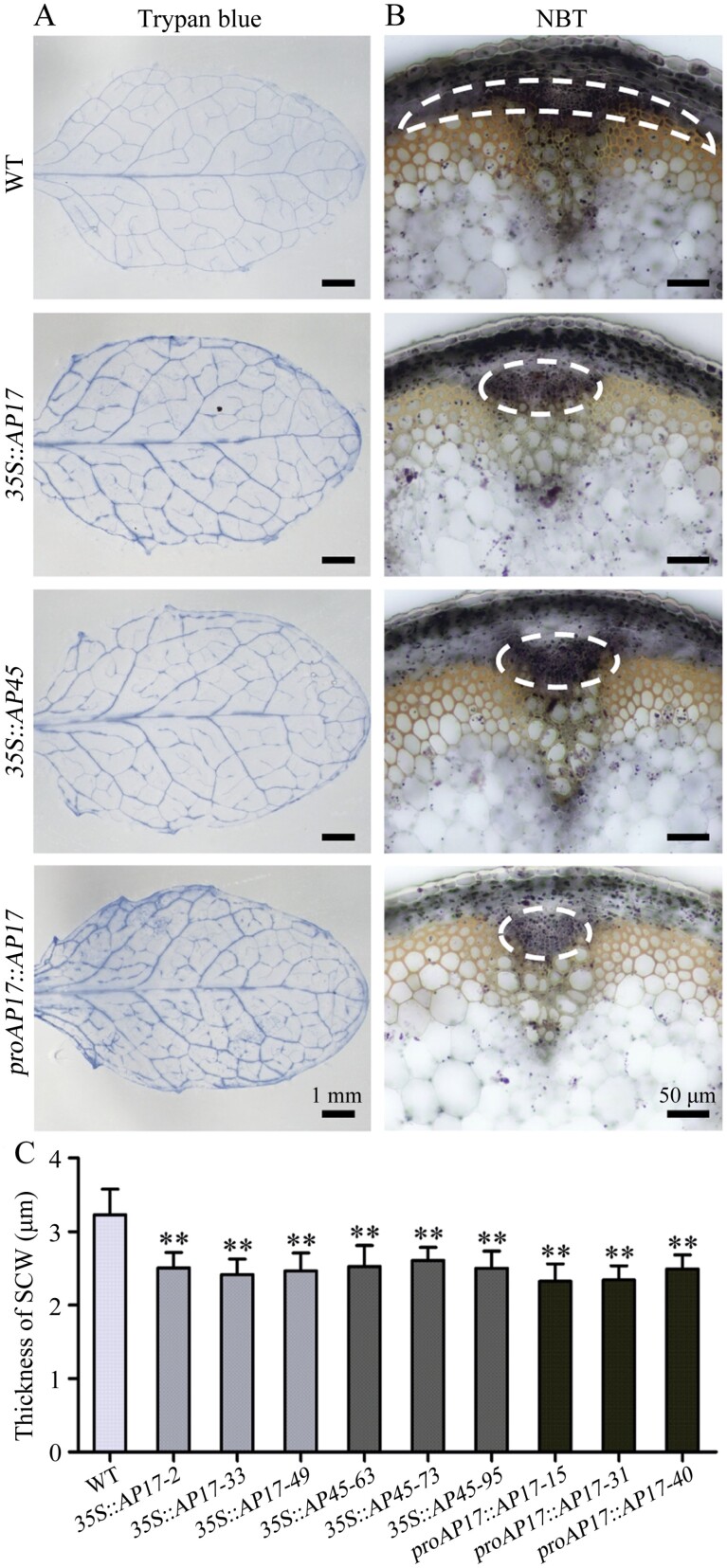
Overexpression of *AP17* or *AP45* in *Arabidopsis* affects PCD and SCW synthesis in transgenic plants. (A) Trypan blue staining analysis of 3-week-old leaves from WT and *35S::AP17*, *35S::AP45* and *proAP17::AP17* overexpressing plants. Black-blue indicates cell death in tissues. (B) Viability staining analysis of metaxylem and the interfascicular fibres (IFs) in the basal stems of WT, and *35S::AP17*, *35S::AP45*, and *proAP17::AP17* overexpressing plants when the leaves are fully senescent. Living cells are surrounded by white dotted line as indicated. (C) Wall thickness of Ifs in WT, *35S::AP17-2/33/49*, *35S::AP45-63/73/95*, and *proAP17::AP17-15/31/40* overexpressing plants. Values are means ±SD (n=15) from three biological repeats. Asterisks denote significant differences from the WT by one-way ANOVA (**P<0.01).

To further confirm the roles of *AP17* or *AP45* in PCD and SCW synthesis, we ectopically overexpressed *AP17* or *AP45* driven by the *35S* promoter or *proAP17* in Arabidopsis. Compared with the WT, the obtained overexpression lines (Supplementary Fig. S11) did not display any visible morphological alterations in overall plant growth. However, stronger trypan blue staining signals were observed in the leaf veins of these overexpression lines than those in WT plants (Fig. 6A), indicating premature cell death in leaf vascular tissues promoted by *AP17* or *AP45* overexpression. Meanwhile, the IFs in the overexpression lines died prematurely (Fig. 6B). Statistical data showed that, compared with the WT, the wall thickness of IFs decreased by 22.3–25.2%, 19.2–22.5%, and 22.8–28.0% in the *35S::AP17*, *35S::AP45*, and *proAP17::AP17* lines (Fig. 6C; Supplementary Fig. S12). Overall, these findings indicate that ectopic overexpression of *AP17* or *AP45* accelerates PCD and reduces the wall thickness of IFs in Arabidopsis.

Plant APs generally contain two conserved aspartic residues crucial for protease activity, and the D^106^ and D^293^ of AP17 and AP45 are conserved with other typical APs from different species (Supplementary Fig. S1B). To determine whether the activity of AP17 or AP45 was essential for its biological function, we examined the effects of *AP17*^*D106/293N*^*-* and *AP45*^*D106/293N*^-mutated variants on PCD and SCW thickness in their overexpression lines in Arabidopsis (Supplementary Fig. S13). Trypan blue staining signals in leaf veins, vascular cell viability, and IF wall thickness in these variants were similar to those in WT plants (Supplementary Figs S14, S15). However, our data suggest that overexpression of *AP17* or *AP45* accelerates PCD and reduces SCW thickness of IFs in Arabidopsis (Fig. 6). Therefore, these results indicate that AP activities of AP17 and AP45 are essential for their roles in PCD and SCW synthesis *in vivo*.

### Saccharification yield in wood is enhanced by perturbation of *AP17/AP45* expression in *P. trichocarpa*

**Fig. 7.**
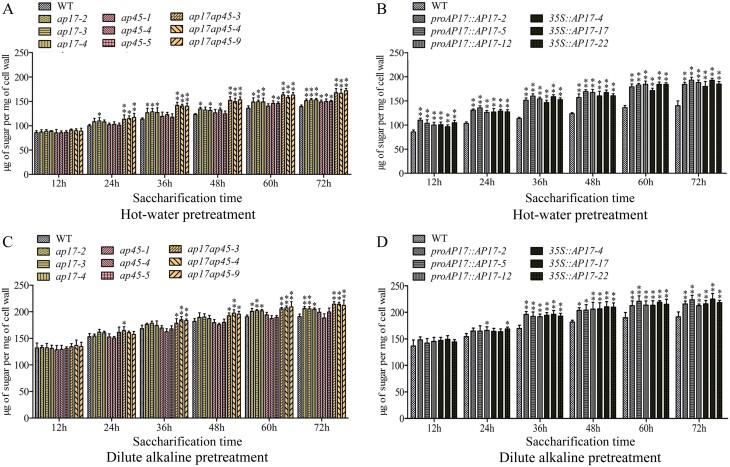
Analysis of enzymatic saccharification in wood cell walls of *AP17/45*-engineered plants. The amount of sugars released from enzymatic digestion (after 12, 24, 36, 48, 60, or 72 h) of wood cell walls derived WT, *ap17*, *ap45*, and *ap17ap45* mutants, and *proAP17::AP17* and *35S::AP17* transgenic lines after hot water (A, B) or dilute alkaline (C, D) pre-treatments, respectively. Values are means ±SD (n=3) from three biological replicates. Asterisks denote significant differences from the WT by one-way ANOVA (*P<0.05; **P<0.01).

As genetic perturbation of *AP17*/*45* alters wood SCW synthesis in *P. trichocarpa* (Fig. 5), we tested whether these transgenic plants could be applied to biofuel utilization. Wood cell wall digestibility was examined in the WT, *AP17*-overexpressing plants, and the *ap17*, *ap45*, and *ap17ap45* mutants. The wood cell wall powders of these materials were mildly pre-treated with hot water or dilute alkali, and the amount of liberated sugars was measured after enzymatic saccharification for 12, 24, 36, 48, 60, and 72 h (Fig. 7). At each time point, sugar release from the wood cell walls of transgenic plants was faster than that from WT plants. For example, saccharification yields at 72 h after hot water pre-treatment were increased by 6.3–12.2% in *ap17* lines, 3.6–10.8% in *ap45* lines, and 14.9–27.3% in *ap17ap45* lines (Fig. 7A). More prominently, a significant increase in saccharification yield after hot water pre-treatment was increased by 27.7–40.8% in *proAP17::AP17* plants, and a 21.5–40.6% increase in *35S::AP17* plants (Fig. 7B). These data showed that saccharification yields in *AP17* overexpressing plants were increased significantly. In addition, saccharification yields after dilute alkali pre-treatment were also increased in *AP17*-overexpressing plants and *ap17ap45* mutants (Fig. 7C, D). Overall, these findings indicate that genetic perturbation of *AP17* and *AP45* expression could enhance the saccharification efficiency of wood cell walls in *P. trichocarpa*.

## Discussion

### *AP17* and *AP45* of typical APs positively modulate xylem maturation in wood formation and are proposed as indicators of wood fibre PCD

Genetic evidence of secondary xylem maturation, especially PCD, is vital for understanding wood formation in angiosperm trees. Our findings showed that loss of *AP17* and/or *AP45* maintains secondary xylem fibres of *ap17*, *ap45*, *ap17ap45* mutants alive for longer (Fig. 3), indicating that *AP17*/*45* plays a positive role in wood fibre maturation. No delay in secondary xylem vessel cell death was observed in these mutants. This is supported by the fact that *AP17* and *AP45* are dominantly expressed in the fibres, not TEs, of secondary xylem, as suggested by their promoter activities (Fig. 1). CEP1 is a papain-like cysteine protease with a KDEL ER retention signal and expressed in various tissues of Arabidopsis, which was involved in tapetal PCD and xylem fibre PCD (Zhang *et al.*, 2014b; Han *et al.*, 2019). In contrast to the location of the CEP1, AP17 and AP45 contain PSI domains (as a vacuolar sorting signal), suggesting a distinct role in fibre maturation. Furthermore, overexpression of *AP17* accelerated secondary xylem fibre cell death in transgenic poplars, and ectopic overexpression of *AP17* likewise promoted IF cell death in Arabidopsis (Figs 4, 6). Together, AP17/45 of typical APs are a positive modulator of xylem maturation, especially fibre PCD, during wood formation.

Morphological changes indicate that xylem fibres of *Populus* stems have a unique cell death programme, different from the pattern of xylem TE maturation (Courtois-Moreau *et al.*, 2009). We determined whether the differences occur in the post-mortem cellular clearance of the fibres in the WT and the *ap17ap45* mutants. However, we failed to obtain reliable TEM data for this aspect. Alternatively, Coomassie staining of xylem elements assessing the remnants of cell contents indicate that the *ap17*, *ap45*, and *ap17ap45* mutants delay fibre cell death during wood formation, and overexpression of *AP17* advances xylem fibre cell death in transgenic plants (Figs 3, 4). As shown in the comparative developing xylem transcriptome, many misregulated genes with low expression in the CA-CE were upregulated in the *ap17ap45* mutants, and conversely, a number of SCW formation and maturation-related genes were downregulated (Supplementary Fig. S7B). This suggests that loss of *AP17* and *AP45* prolongs the development progress before secondary xylem cell death, agreeing with the data that the thickness of the developing fibre walls reduces in the *ap17ap45* mutants (Supplementary Fig. S10). Thus, *AP17* and *AP45* genes could be proposed as indicators of the fibre PCD during wood formation based on their roles and expression levels.

Most recently, a review has summarized molecular events in wood PCD (Luo and Li, 2022), but current understanding of this aspect is quite insufficient. AP17/45 contains the PSI domain and C-terminal FAEA (Supplementary Fig. S1B), suggesting its location in the vacuole and its provacuolar compartment (PVC). The protein containing the FAEA peptide is likely sorted via the coat protein complex II-mediated ER-Golgi-PVC pathway (Pereira *et al.*, 2013). Our data (Figs 3–5; Supplementary Fig. S7B) imply that *AP17/45* is involved in differentiation-induced dPCD in xylem fibre maturation. As xylem fibre PCD is initiated ahead of vacuolar disintegration (Courtois-Moreau *et al.*, 2009), it is proposed that *AP17/45* modulates wood fibre PCD, probably via a vacuole non-destructive way, different from the vacuolar-collapse system (Hara-Nishimura and Hatsugai, 2011). For example, there is a possibility that some proteins in the vacuole and/or PVC modified by AP17/45 could flow out and regulate differentiation-induced dPCD in xylem fibres. Identification of the substrates of AP17/45 will help us further understand this process during wood formation.

### Perturbation of *AP17*/*45* alters wood SCW synthesis in poplar, improving enzymatic saccharification yields for biofuel production

*AP17*/*45* modulates wood SCW synthesis in *P. trichocarpa*, supported by the reduced thickness of fibre SCWs in *AP17* overexpression poplars and ectopic *AP17*/*45* overexpression in Arabidopsis plants (Figs 5, 6). A decrease in lignin content in wood was also found in the *ap17*, *ap45i*, and *ap17ap45* mutants, most probably due to the delay in post mortem lignification of the fibres because of the prolonged lifetime. It is in line with earlier hypotheses on the requirement of cell death for full lignification of the xylem elements. Transcriptome data further suggested the involvement of AP17/45 in wood SCW synthesis (Supplementary Fig. S7; Supplementary Table S5). Additionally, SNBE, SMRE, and M46RE *cis*-elements were found in the promoter regions of *AP17* and *AP45* (Cao *et al.*, 2019). These *cis*-elements are involved in SCW formation at the transcriptional level (Zhong *et al.*, 2010, 2012). Gene expression analysis revealed significant overlap in the control of fibre SCW formation and PCD in *Populus* stems (Courtois-Moreau *et al.*, 2009). Our findings provide genetic and wood chemical evidence for the function of *AP17*/*45* in wood fibre PCD and SCW formation in poplar. Some evidence demonstrates that TE PCD is partly coupled with SCW formation, as suggested by direct regulation of *VND6* or *XND1* in some SCW- and PCD-related genes and an overlapping inhibition of SCW and PCD by pharmacological treatment (Yamamoto *et al.*, 1997; Groover and Jones, 1999; Zhao *et al.*, 2008; Ohashi-Ito *et al.*, 2010). Thus, an overlap in the SCW and PCD processes occurs in wood fibre maturation.

Poplar, an important bioenergy crop, provides a large amount of lignocellulosic biomass for the generation of biofuels. Our data suggest that *AP17* and *AP45* are potential candidate genes for engineering lignocellulosic wood. A 14.9–27.3% increase in saccharification yield was observes in the *ap17ap45* mutant woods (Fig. 7); this is ascribed to a significant decrease in lignin content, as supported by the downregulation of lignin biosynthetic genes (Supplementary Tables S4, S5). Lignin is the main factor limiting the enzymatic hydrolysis of cellulose into glucose from lignocellulosic biomass, and the decreased lignin content is beneficial for biomass conversion; for example, the knockdown of 4-coumarate:coenzyme A ligase, cinnamoyl-CoA reductase, or caffeoyl shikimate esterase leads to reduced lignin content and improved sugar yields for biofuel production (Xu *et al.*, 2011; Van Acker *et al.*, 2014; Jang *et al.*, 2021). The *AP17*-overexpressed poplars also showed a 21.5–40.8% increase in the saccharification yield (Fig. 7). Together with the reduced thickness of wood fibre SCWs (Fig. 5), this might be associated with reduced lignin content and/or alteration of lignin structure in immature SCWs caused by premature PCD. Additionally, no biomass penalty is required to improve the enzymatic saccharification efficiency in poplars through modified lignin biosynthesis. Suppression of lignin synthesis in vessels causes growth defects and substantial reductions in biomass yield in poplar (Gui *et al.*, 2020). *AP17* overexpression poplars and *ap17ap45* mutants displayed no biomass penalty (Figs 3A, 4A). Considering that *AP17*/*45* is dominantly expressed in secondary xylem fibres, alteration of lignin synthesis should mainly occur in the wood fibres of these transgenic poplars.

In summary, *AP17* and *AP45* of *P. trichocarpa* typical APs positively modulate xylem maturation, mainly fibre PCD, in wood formation. Additionally, *AP17* and *AP45* are involved in SCW synthesis during wood formation. Valuably, alterations in *AP17*/*45* expression lead to improved saccharification yield in the wood of transgenic poplars, providing a candidate gene for engineering lignocellulosic wood for biofuel utilization.

## Supplementary data

Supplementary data are available at *JXB* online.

Table S1. All primers used in this study.

Table S2. The sequences of typical APs from different species.

Table S3. *AP17* and/or *AP45* mutations by the Cas9/gRNA in multiple transgenic lines.

Table S4. The DEGs of developing xylem in the *ap17ap45* mutants compared with WT plants.

Table S5. Expression data for lignin synthesis genes in the *ap17ap45* mutants compared with WT plants.

Fig. S1. Characterization of *P. trichocarpa* typical APs.

Fig. S2. Tissue expression activities of *AP19*, *AP42*, and *AP47* gene promoters in *P. trichocarpa*.

Fig. S3. Expression profiles of typical *AP* genes in *Populus* stem tissues.

Fig. S4. Characterization of the *ap17*, *ap45*, and *ap17ap45* mutants.

Fig. S5. Evans blue staining of dead vessels in WT and transgenic plant stem xylem.

Fig. S6. Verification of the DEGs by RT-qPCR analysis in the WT and the *ap17ap45* mutant.

Fig. S7. Transcriptome analysis of developing xylem in the WT and the *ap17ap45* mutant.

Fig. S8. Expression levels of *AP17* in the developing xylem of two types of overexpressing plants.

Fig. S9. Characterization of the *35S::AP17* and *proAP17::AP17* overexpressing plants.

Fig. S10. Wall thickness of the developing (immature) fibres in the *ap17ap45* mutants.

Fig. S11. Expression levels of *AP17* and *AP45* in overexpressing Arabidopsis plants.

Fig. S12. Wall thickness of interfascicular fibres in the WT, and the *35S::AP17*, *35S::AP45*, and *proAP17::AP17* overexpressing plants.

Fig. S13. Expression levels of *AP17*^*D106/293N*^ and *AP45*^*D106/293N*^ in overexpressing Arabidopsis plants.

Fig. S14. Gain-of-function of *AP17* or *AP45* depends on two conserved aspartic acid residues (D^106/293^).

Fig. S15. Wall thickness of interfascicular fibres in the WT, the *35S::AP17*^*D106/293N*^*-26/51/76*, *35S::AP45*^*D106/293N*^*-32/55/63*, and *proAP17::AP17*^*D106/293N*^*-19/43/52* overexpressing plants.

erac347_suppl_Supplementary_Figures_S1-S15_Tables_S1-S5

## Data Availability

All data supporting the findings of this study are available within the paper and within its supplementary data published online. The sequences from this article can be found in Phytozome (version 13.0) under the following accessions: *AP6* (Potri.001G356900), *AP11* (Potri.002G228300), *AP17* (Potri.004G007600), *AP19* (Potri.005G002800), *AP42* (Potri.010G003400), *AP45* (Potri.011G007600), and *AP47* (Potri.013G002200).
