# Supplementary material for: Aspartic proteases modulate programmed cell death and secondary cell wall synthesis during wood formation in poplar
Source: J Exp Bot. 2022 Aug 30;73(19):6876–90. doi: 10.1093/jxb/erac347 (PMC9629783; doi:10.1093/jxb/erac347)
Supplement: erac347_suppl_Supplementary_Figures_S1-S15_Tables_S1-S5 [file erac347_suppl_supplementary_figures_s1-s15_tables_s1-s5.pdf]

Aspartic proteases modulate programmed cell death and secondary cell wall synthesis during wood formation in poplar

Supplementary information:

Fig. S1. Characterization of *P. trichocarpa* typical APs.
Fig. S2. Tissue expression activities of *AP19*, *AP42*, and *AP47* gene promoters in *P. trichocarpa*.
Fig. S3. Expression profiles of typical *AP* genes in *Populus* stem tissues.
Fig. S4. Characterization of the *ap17*, *ap45*, and *ap17ap45* mutants.
Fig. S5. Evans blue staining of dead vessels in wild-type and transgenic plant stem xylem.
Fig. S6. Verification of the DEGs by RT-qPCR analysis in wild-type and *ap17ap45* mutants.
Fig. S7. Transcriptome analysis of developing xylem in wild-type and *ap17ap45* mutants.
Fig. S8. Expression levels of *AP17* in developing xylem of two types of overexpression plants.
Fig. S9. Characterization of the *35S::AP17* and *proAP17::AP17* overexpression plants.
Fig. S10. Wall thickness of the developing (immature) fibers in the *ap17ap45* mutants.
Fig. S11. Expression levels of *AP17* and *AP45* in their overexpression *Arabidopsis* plants.
Fig. S12. Wall thickness of interfascicular fibers in wild-type, *35S::AP17*, *35S::AP45*, and *proAP17::AP17* overexpression plants.
Fig. S13. Expression levels of $AP17^{D106/293N}$ and $AP45^{D106/293N}$ in their overexpression *Arabidopsis* plants.
Fig. S14. Gain-of-function of *AP17* or *AP45* depends on the two conserved aspartic residues ($D^{106/293}$).
Fig. S15. Wall thickness of interfascicular fibers in wild-type, $35S::AP17^{D106/293N}$*-26/51/76*, $35S::AP45^{D106/293N}$*-32/55/63*, and $proAP17::AP17^{D106/293N}$*-19/43/52* overexpression plants.

Table S1. All primers used in this study.
Table S2. The sequences of typical APs from different species.
Table S3. *AP17* and/or *AP45* mutations by the *Cas9*/gRNA in multiple transgenic lines.
Table S4. The DEGs of developing xylem in the *ap17ap45* mutants compared with wild-type plants.
Table S5. Expression data for lignin synthesis genes in the *ap17ap45* mutants compared with wild-type plants.

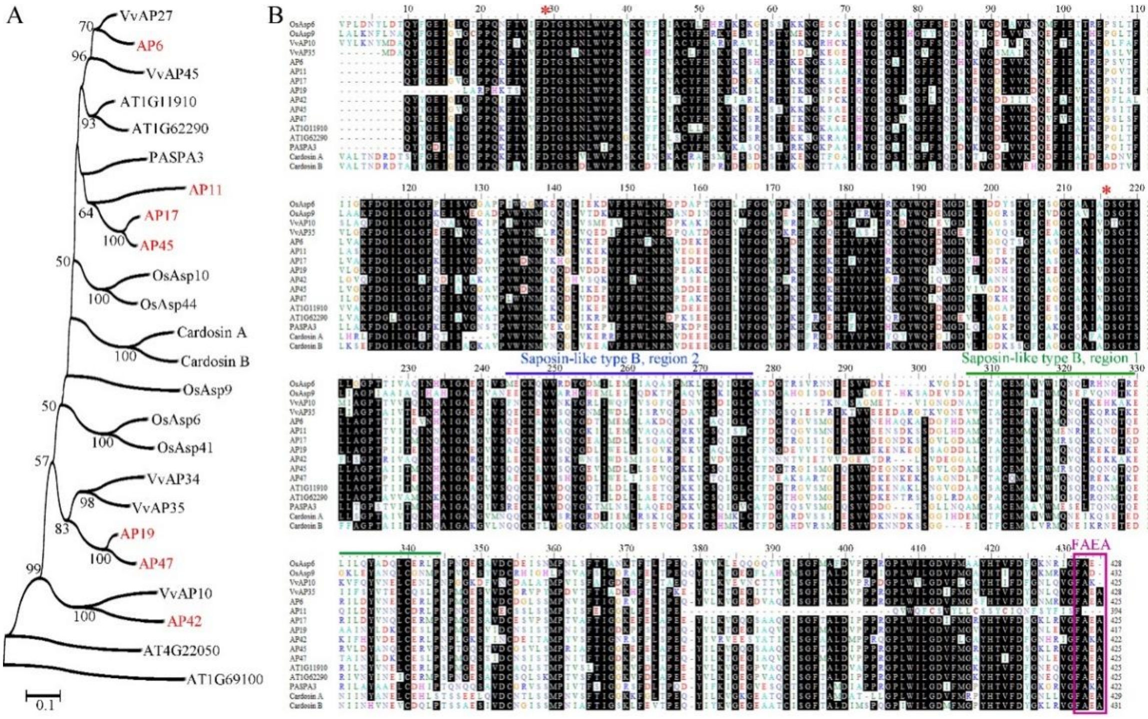

Fig. S1. Characterization of *P. trichocarpa* typical APs. (A) Phylogenetic tree of typical APs from *P. trichocarpa*, *Arabidopsis* (Faro & Gal, 2005), grape (Guo *et al.*, 2013) and rice (Chen *et al.*, 2009). Relationships were based upon amino acid similarity and generated using Clustal X (Larkin *et al.*, 2007) and MEGA7 (Kumar *et al.*, 2016). The tree was constructed under poission model by the neighbor-joining method with 1000 bootstrap replications. *Populus* APs are denoted by the red letters. Cardosin A (Ramalho-Santos *et al.*, 1998) and Cardosin B (Vieira *et al.*, 2001) are included to show its relationship to these typical APs. The scale bar indicates the number of amino acid substitution per site. (B) Multiple sequences alignment of ASP domain (PF00026) of typical APs from different species. The red asterisks show the conserved aspartic catalytic residues. The lines at the top of the sequences indicate the SapB_1 (Saposin-like type B, region 1) and SapB_2 domain, respectively. The C-terminal FAEA motif is marked by pink box. Identical amino acid residues are denoted by black letters on a white background.

References

Chen J, Ouyang Y, Wang L, Xie W, Zhang Q. 2009. Aspartic proteases gene family in rice: Gene structure and expression, predicted protein features and phylogenetic relation. *Gene* **442,** 108-118.

Guo R, Xu X, Carole B, *et al*. 2013. Genome-wide identification, evolutionary and expression analysis of the aspartic protease gene superfamily in grape. *BMC Genomics* **14,** 554.

Faro C, Gal S. 2005. Aspartic proteinase content of the Arabidopsis genome. *Current protein & peptide science* **6,** 493-500.

Kumar S, Stecher G, Tamura K. 2016. MEGA7: Molecular evolutionary genetics analysis version 7.0 for bigger datasets. *Molecular Biology and Evolution* **33,** 1870-1874.

Larkin MA, Blackshields G, Brown NP, *et al.* 2007. Clustal W and Clustal X version 2.0. *Bioinformatics* **23,** 2947-2948.

Vieira M, Pissarr J, Veríssimo P, *et al*. 2001. Molecular cloning and characterization of cDNA encoding cardosin B, an aspartic proteinase accumulating extracellularly in the transmitting tissue of *Cynara cardunculus* L. *Plant Molecular Biology* **45,** 529-539.

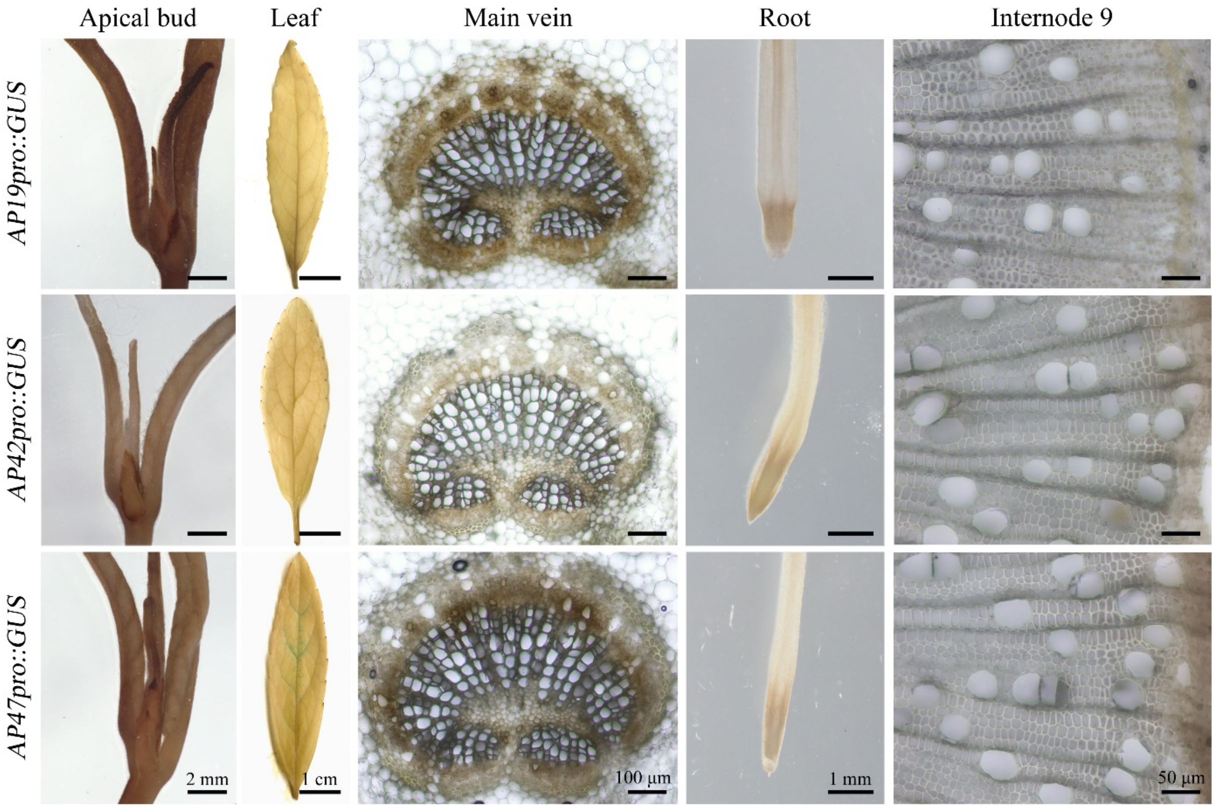

Fig. S2. Tissue expression activities of *AP19, AP42,* and *AP47* gene promoters in *P. trichocarpa*. GUS activity driven by typical *AP19*, *42*, or *47* gene promoter was analyzed in corresponding *APpro::GUS* transgenic *P. trichocarpa*, respectively. Leaf and root from 4-week-old transgenic plants; apical bud, main vein and stem from 3-month-old transgenic plants.

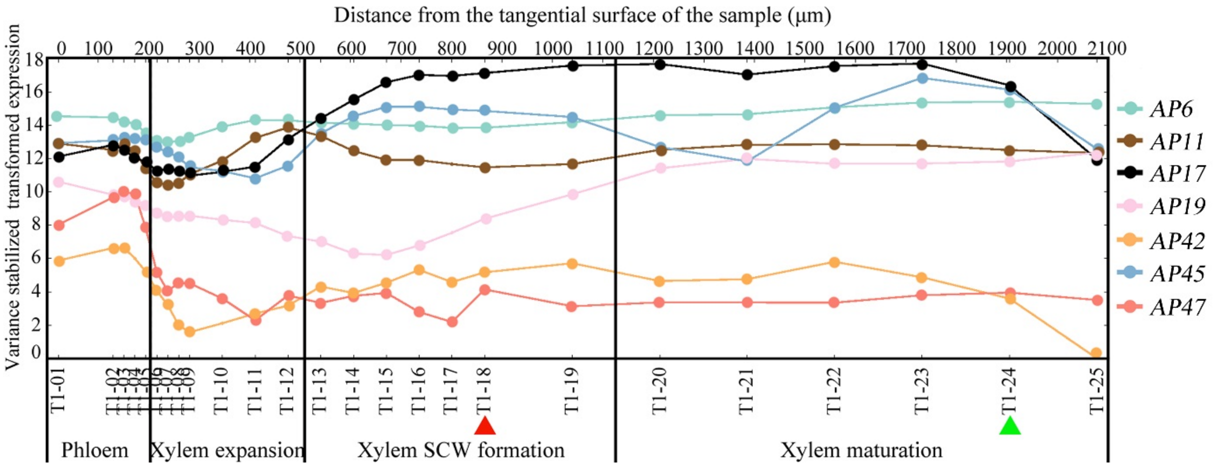

Fig. S3. Expression profiles of typical AP genes in *Populus* stem tissues. Gene expression of typical APs is shown across the stem secondary tissues derived from the AspWood database (http://aspwood.popgenie.org). Tissue samples 01-25 are shown below while the physical distances are shown above the graph. The red and green arrows indicate the approximate locations of vessel and fiber cell death as described previously (Sundell *et al.*, 2017).

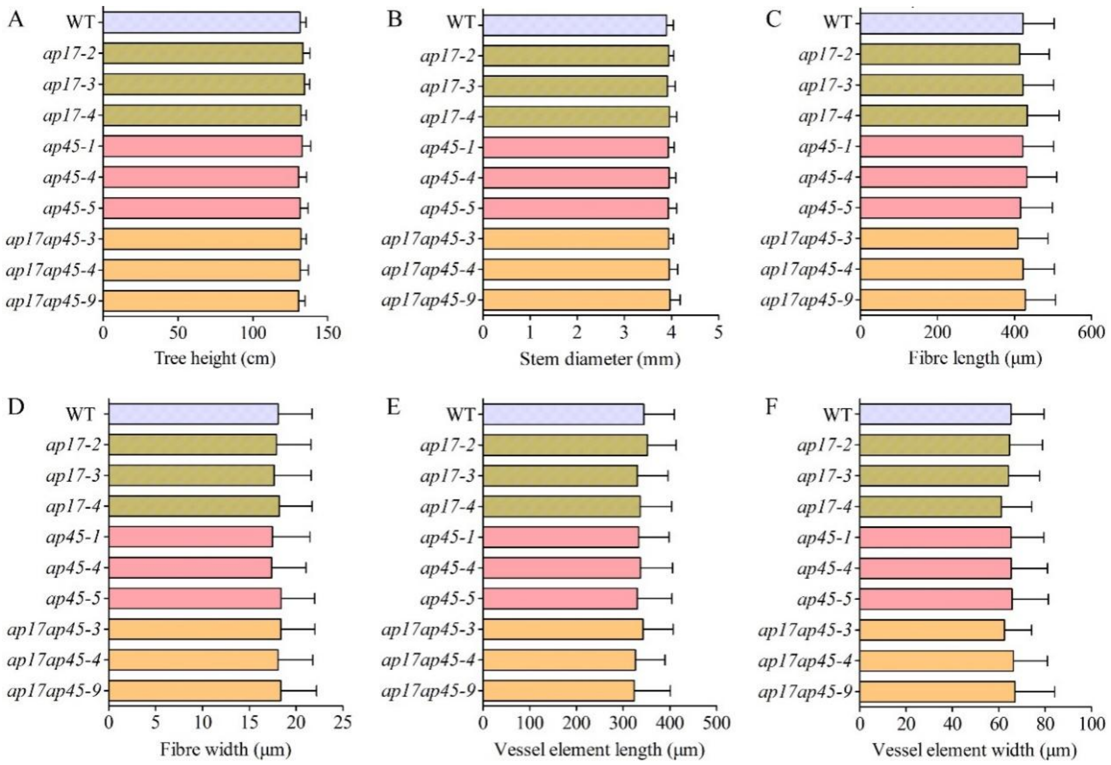

Fig. S4. Characterization of the *ap17, ap45* and *ap17ap45* mutant plants. The wild-type (WT), *ap17*, *ap45* and *ap17ap45* mutants were grown in the greenhouse for 4 months, and measurement for trees height (A), stems diameter (B), length of the xylem fibers (C), radial width of the xylem fibers (D), length of the xylem vessels (E), and radial width of the xylem vessels (F). Values are means ± SD from four biological replicates.

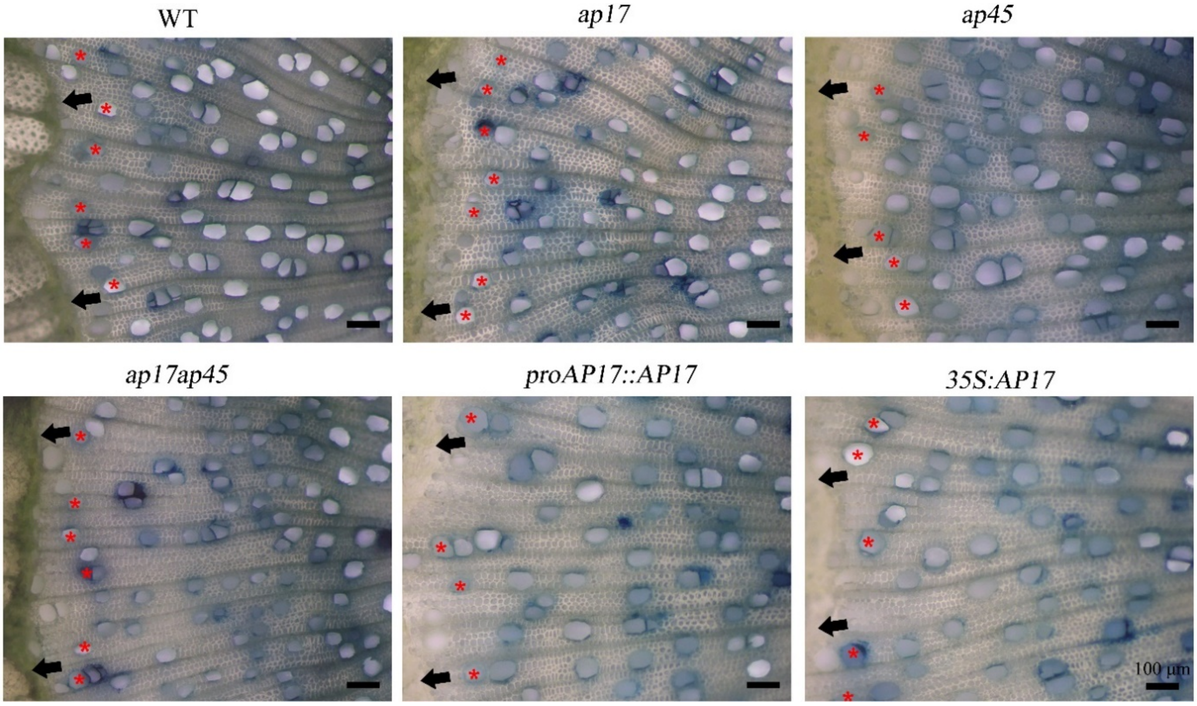

Fig. S5. Evans blue staining of dead vessels in wild-type and transgenic plant stem xylem. Images are shown for transverse sections of the 15th stem internodes from 4-month-old wild-type (WT) and transgenic trees. The staining reveals that the vessel elements die and start transporting water within the similar distances from the cambium in this case. The asterisk indicates a recently functionalized vessel element, and arrowheads indicate vascular cambium.

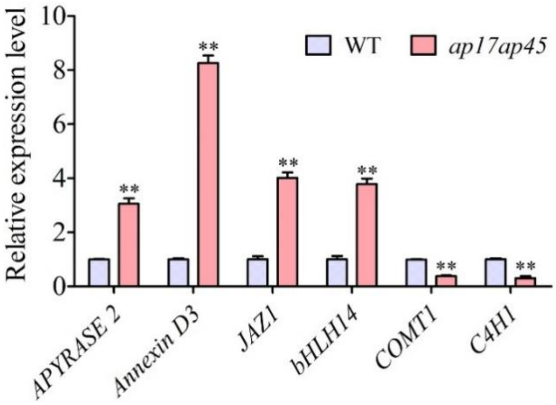

Fig. S6. Verification of the DEGs by RT-qPCR analysis in wild-type and *ap17ap45* mutants. Expression profiles of the 6 DEGs (*APYRASE 2*, *Annexin D3*, *JAZ1*, *bHLH14*, *COMT1* and *C4H1*) was verified in *ap17ap45* and wild-type plants. Relative expression levels were normalized using the geometric mean of *PtActin2*, *PtEF1β* and *PteIF5A* expression. Error bars represent SD values from three biological repeats. Significance test was conducted by one-way ANOVA (**$P < 0.01$).

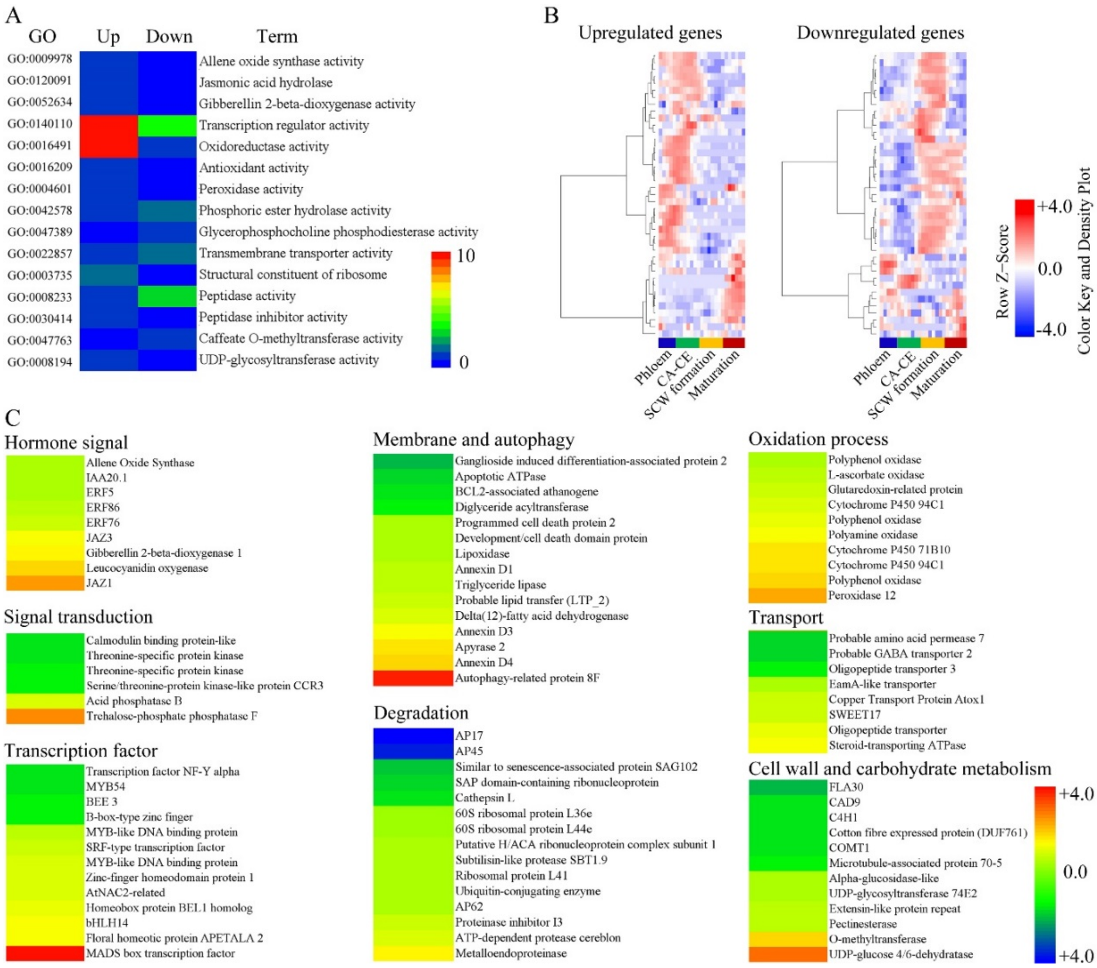

Fig. S7. Transcriptome analysis of developing xylem in wild-type and *ap17ap45* mutants. (A) GO enrichment analysis of DEGs. (B) Heatmaps show transcription profiling of the DEGs in wood tissues using the AspWood database (aspwood.popgenie.org). CA-CE represents cambium and cell expansion. (C) Classification of most DEGs according to gene annotations or known functions of the homologues. The expression ratios (log2 values) are indicated by red/ blue colored squares.

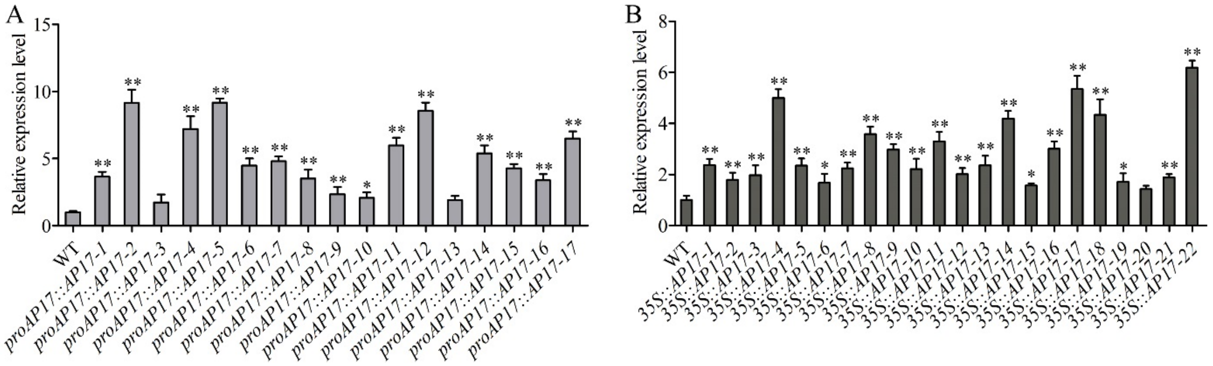

Fig. S8. Expression levels of *AP17* in developing xylem of two types of overexpression plants. (A) Expression levels of *AP17* in *35S::AP17* transgenic *P. trichocarpa* lines. (B) Expression levels of *AP17* in *proAP17::AP17* transgenic *P. trichocarpa* lines. Relative expression levels were normalized using the geometric mean of *Ptactin2*, *PtEF1β* and *PteIF5A* expression. Error bars represent SD values from three biological repeats. Significance test was conducted by one-way ANOVA (*$P < 0.05$ and **$P < 0.01$).

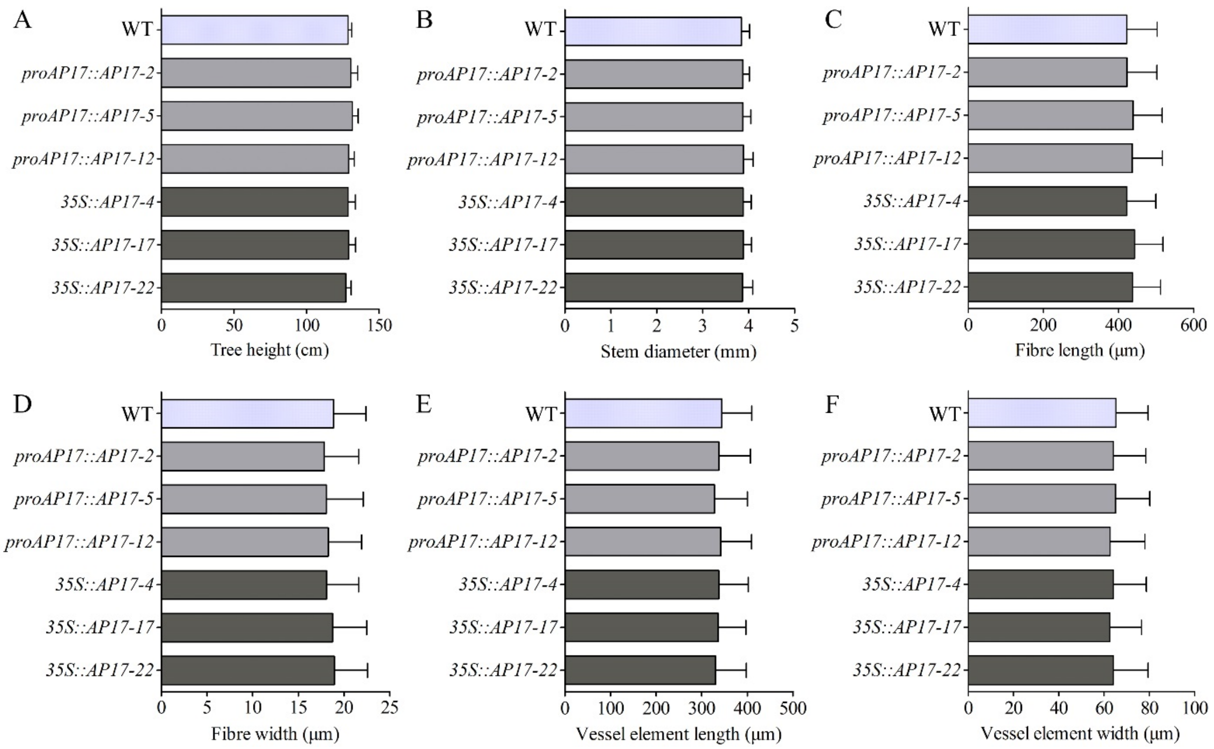

Fig. S9. Characterization of the *35S::AP17*, and *proAP17::AP17* overexpression plants. The *35S::AP17* and *proAP17::AP17* transgenic *P. trichocarpa* lines were grown in the greenhouse for 4 months, and measurement for trees height (A), stems diameter (B), length of the xylem fibers (C), radial width of the xylem fibers (D), length of the xylem vessels (E), and radial width of the xylem vessels (F). Values are means ± SD from four biological replicates.

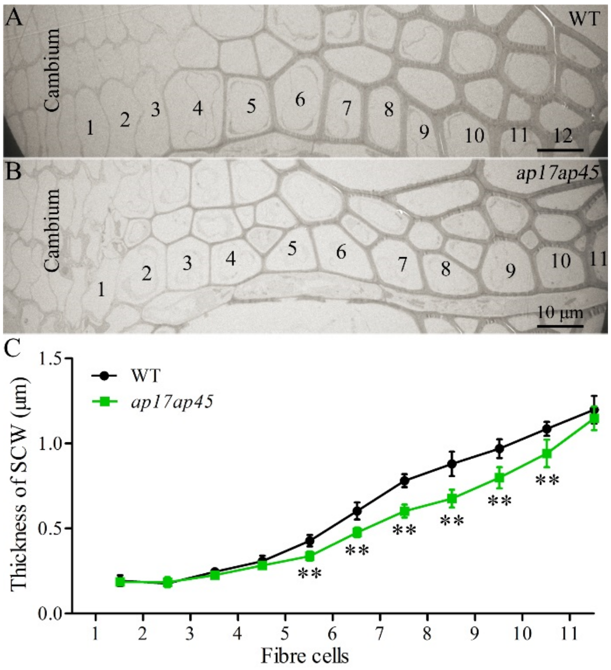

Fig. S10. Wall thickness of the developing (immature) fibers in the *ap17ap45* mutants. (A-B) Developing fiber cells were recorded from wild-type (WT) and *ap17ap45* mutants using a transmission electron microscope. (C) Wall thickness of developing fiber cells. Successive 12 developing cells from each position in wild-type and *ap17ap45* mutants were measured. Values are means ± SD (n =5). Significance test was conducted by one-way ANOVA (***P* < 0.01).

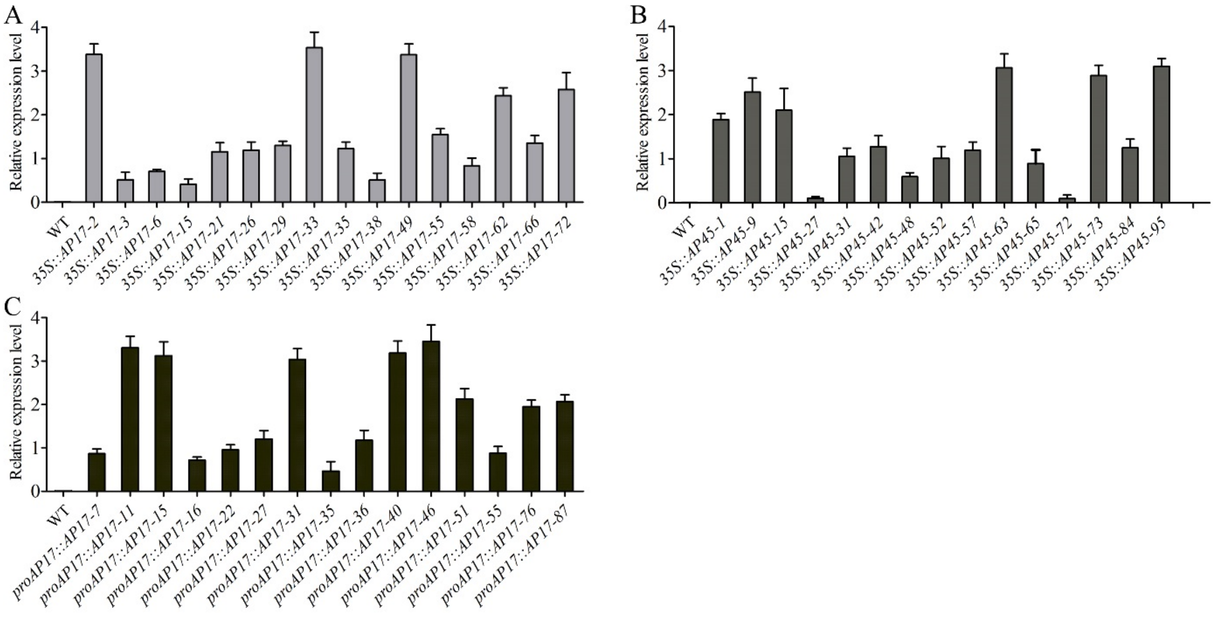

Fig. S11. Expression levels of *AP17* and *AP45* in their overexpression *Arabidopsis* plants. (A) Expression levels of *AP17* in *35S::AP17* transgenic lines. (B) Expression levels of *AP45* in *35S::AP45* transgenic lines. (C) Expression levels of *AP17* in *proAP17::AP17* transgenic lines. The comparative Ct ($2^{-\Delta Ct}$) method was used to calculate transcript abundance, and gene expression levels were normalized using the geometric mean of *Atactin2*, *AtGAPDH* and *AtUBQ10*. Error bars represent SD values from three biological repeats.

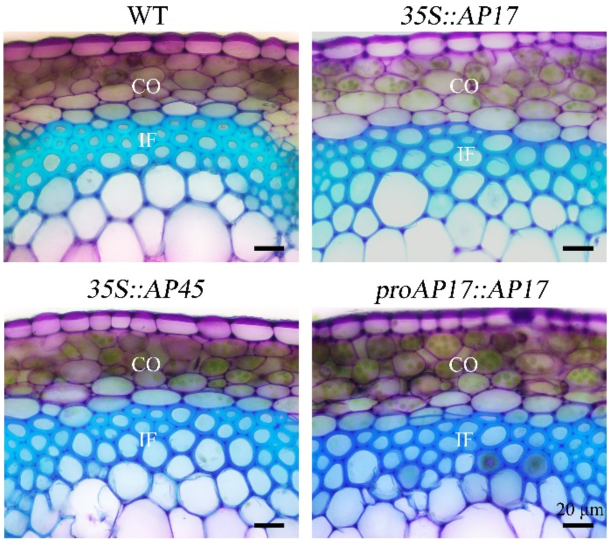

Fig. S12. Wall thickness of IFs in wild-type, *35S::AP17*, *35S::AP45* and *proAP17::AP17* overexpression plants. Wall thickness of the IFs from the stems of wild-type (WT), *35S::AP17*, *35S::AP45*, and *proAP17::AP17* overexpression plants when they finish flowering. Transverse sections of basal stems were stained with toluidine blue. CO, cortex.

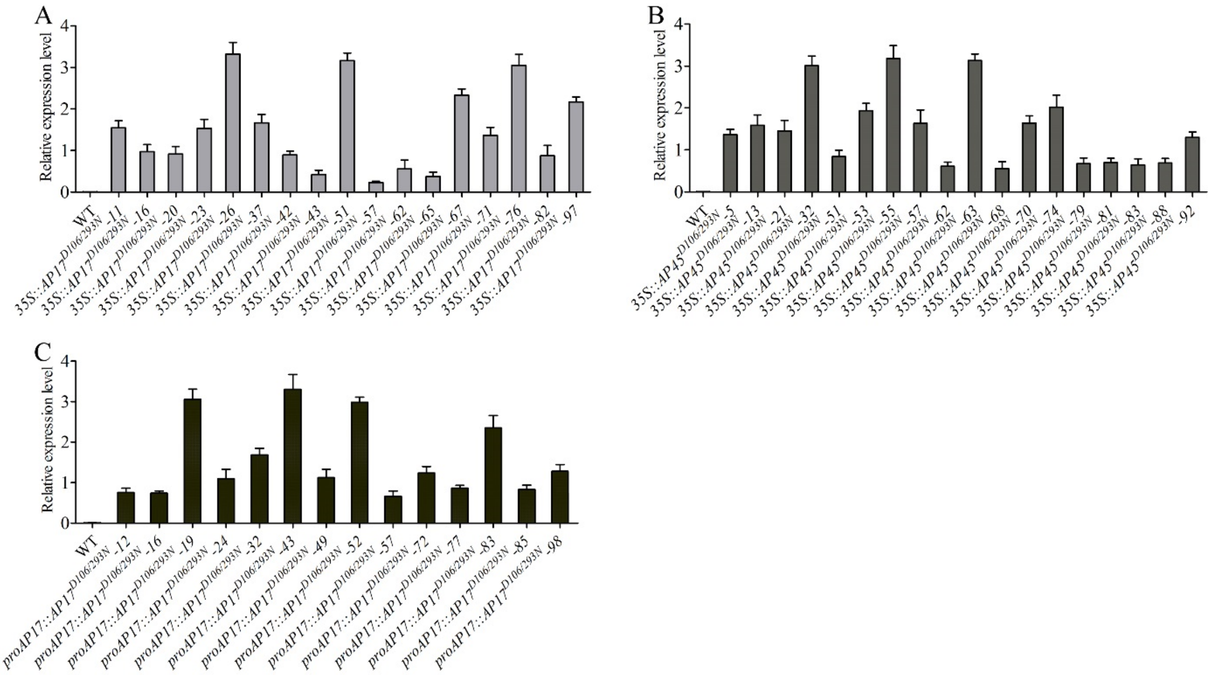

Fig. S13. Expression levels of *AP17*$^{D106/293N}$ and *AP45*$^{D106/293N}$ in their overexpression *Arabidopsis* plants. (A) Expression levels of *AP17*$^{D106/293N}$ in *35S::AP17*$^{D106/293N}$ transgenic lines. (B) Expression levels of *AP45*$^{D106/293N}$ in *35S::AP45*$^{D106/293N}$ transgenic lines. (C) Expression levels of *AP17*$^{D106/293N}$ in *proAP17::AP17*$^{D106/293N}$ transgenic lines. The comparative Ct ($2^{-\Delta Ct}$) method was used to calculate transcript abundance, and gene expression levels were normalized using the geometric mean of *AtActin2*, *AtGAPDH* and *AtUBQ10*. Error bars represent SD values from three biological repeats.

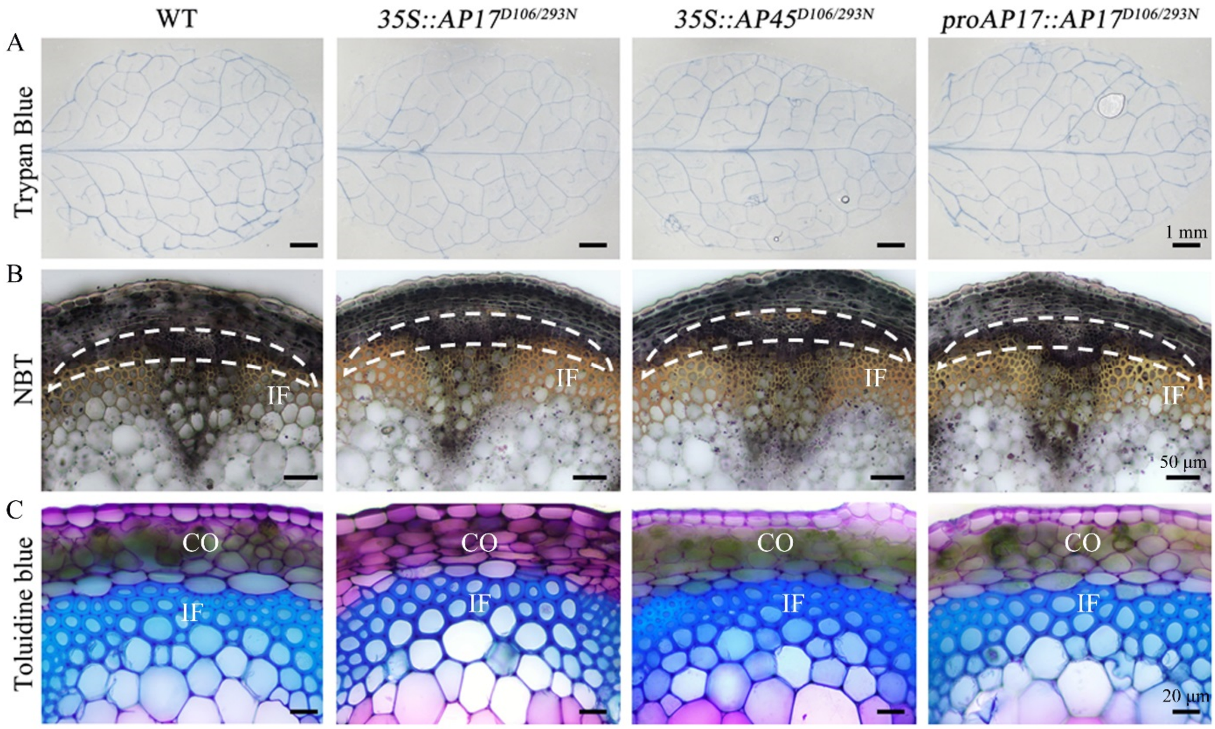

Fig. S14. Gain-of-function of *AP17* or *AP45* depends on the two conserved aspartic residues ($D^{106/293}$). (A) Trypan blue staining analysis of 3-week-old leaves from wild-type (WT), and *35S::AP17*$^{D106/293N}$, *35S::AP45*$^{D106/293N}$, and *proAP17::AP17*$^{D106/293N}$ overexpression plants. (B) Viability staining analysis of metaxylem and IFs in the basal stems of WT and *35S::AP17*$^{D106/293N}$, *35S::AP45*$^{D106/293N}$ and *proAP17::AP17*$^{D106/293N}$ overexpression plants when the leaves are fully senescent. Living cells were surrounded by white dotted line as indicated. (C) Wall thickness of the IFs from WT, *35S::AP17*$^{D106/293N}$, *35S::AP45*$^{D106/293N}$, and *proAP17::AP17*$^{D106/293N}$ overexpression plants when they finish flowering. Transverse sections of basal stems were stained with toluidine blue. CO, cortex.

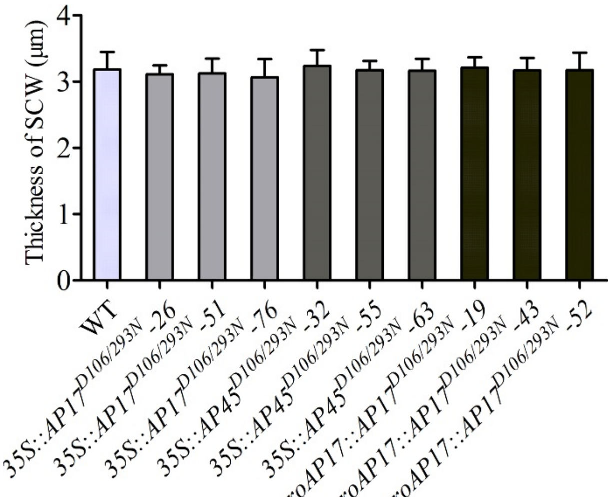

Fig. S15. Wall thickness of interfascicular fibers in wild-type, *35S::AP17$^{D106/293N}$-26/51/76, 35S::AP45$^{D106/293N}$-32/55/63,* and *proAP17::AP17$^{D106/293N}$-19/43/52* overexpression plants. Wall thickness of the IFs from the stems of wild-type (WT), *35S::AP17 $^{D106/293N}$*, *35S::AP45 $^{D106/293N}$*, and *proAP17::AP17 $^{D106/293N}$* overexpression plants when they finish flowering. Error bars represent SD values (n =15) from three biological replicates.

Table S1. All primers used in this study.

Purpose	Primer Name	Sequence (5'-3')
Primers for promoter vector construction		
ProAP6	ProAP6-F	CACCTTTGAAGCCCACGTGGACATGATC
	ProAP6-R	TTTCTGAAATTTATACACCAAC
ProAP11	ProAP11-F	CACCCAAGCCATCAAAGCTCCATCG
	ProAP11-R	AGCTGAGTCGAGACTGTGAACC
ProAP17	ProAP17-F	CACCCTCGAGATACACGCATACTAACC
	ProAP17-R	GTTTACACCTATAAATTGATGTTAAG
ProAP19	ProAP19-F	CACCGAGCAAGTCATATCAGGCACATC
	ProAP19-R	CCAAATAGTTTTTAAGGGGTAT
ProAP42	ProAP42-F	CACCACTCTTATGGAAGCAGCATG
	ProAP42-R	GCTCGATCACAAAAGTTTCTTTC
ProAP45	ProAP45-F	CACCAGACAATCAAGCGCAAACAGTATG
	ProAP45-R	TTTTACACCTGTAAATTCACATTAAC
ProAP47	ProAP47-F	CACCCTAGTTTAGTATCGTCAACGACTCTCC
	ProAP47-R	AGGAGAGAAAAGATACTCCTAG
Primers for CRISPR/Cas9 vector construction		
Cas9/gRNA-AP17-1	DT1-BsF/AP17-1	ATATATGGTCTCGATTGTTAGGAAGTATAATCTTTGGTT
	DT1-F0/AP17-1	TGTTAGGAAGTATAATCTTTGGTTTTAGAGCTAGAAATAGC
	DT2-R0/AP17-1	AACCAGAAGTTCACTGTGATCTCAATCTCTTAGTCGACTCTAC
	DT2-BsR/AP17-1	ATTATTGGTCTCTAAACCAGAAGTTCACTGTGATCTC
Cas9/gRNA-AP17-2	DT1-BsF/AP17-2	ATATATGGTCTCGATTGTATCATCTGCGTCGAATGAGTT
	DT1-F0/AP17-2	TGTATCATCTGCGTCGAATGAGTTTTAGAGCTAGAAATAGC
	DT2-R0/AP17-2	AACGATAATTCTTCAATGCAACCAATCTCTTAGTCGACTCTAC
	DT2-BsR/AP17-2	ATTATTGGTCTCTAAACGATAATTCTTCAATGCAACC
Cas9/gRNA-AP45-1	DT1-BsF/AP45-1	ATATATGGTCTCGATTGTGAACTTGAAAGCAATTGTGTT
	DT1-F0/AP45-1	TGTGAACTTGAAAGCAATTGTGTTTTAGAGCTAGAAATAGC
	DT2-R0/AP45-1	AACGTGTCAAAAATCACTGTGACAATCTCTTAGTCGACTCTAC
	DT2-BsR/AP45-1	ATTATTGGTCTCTAAACGTGTCAAAAATCACTGTGAC

Cas9/gRNA-AP17/AP45-1	DT1-BsF/AP17/AP45-1	ATATATGGTCTCGATTGCTGGAACTTCCTTGTTGGCGTT
	DT1-F0/AP17/AP45-1	TGCTGGAACTTCCTTGTTGGCGTTTTAGAGCTAGAAATAGC
	DT2-R0/AP17/AP45-1	AACAATCATTACAAGGGCAAGCCAATCTCTTAGTCGACTCTAC
	DT2-BsR/AP17/AP45-1	ATTATTGGTCTCTAAACAATCATTACAAGGGCAAGCC
Primers used to identify Cas9-gRNAs targeted sites		
gAP17-a/b/c/d-identify	gAP17- a/b/c/d -identify-F	GGACATTGCATACAGTAGACAAG
	gAP17- a/b/c/d -identify-R	GCTAAACTGTCTTCTCATGGAATG
gAP45-e/f-identify	gAP45-e/f-identify-F	AAGATCATGCAGTGGAGAACTGC
	gAP45-e/f-identify-R	TGATAGCATCGCATCCTTAGTTCC
gAP17-g/h-identify	gAP17-g/h-identify-F	TTGTGTTTGAGTGACACACGTGC
	gAP17-g/h-identify-R	TCCATACAACTTCAACTCAAGGTC
gAP45-g/h-identify	gAP45-g/h-identify-F	TTGTGTTTGAGTTGACATAGTGC
	gAP45-g/h-identify-R	TCAACATAACTTCAACCCAATGGC
Primers for overexpression vector construction		
AP17-B11 (35S)	AP17-F	CACCATGGGAGTGAACTTGAAAGCAATTG
	AP17-R	AGCTGCCTCTGCAAATCCAACTC
AP45-B11 (35S)	AP45-F	CACCATGGGAGTGAACTTGAAAGCAATTG
	AP45-R	CGCTGCCTCGGCAAATCCAACTC
AP17-B10 (native promoter)	AP17PC-F	CATCAATTTATAGGTGTAAACATGGGAGTGAACTTGAAAGCAATTG
	AP17PC-R	CAATTGCTTTCAAGTTCACTCCCATGTTTACACCTATAAATTGATG
Primers for site-directed mutagenesis vector construction		
$AP17^{D106N}$	AP17MutD106N-F	GTGATCTTTAACACTGGTAGCTCCAATTTGTG
	AP17MutD106N-R	AGTGAACTTCTGAGGAGGAGAGCCAACACC
$AP17^{D293N}$	AP17MutD293N-F	CAATTGCAAATTCTGGAACTTCCTTGTTGGC
	AP17MutD293N-R	CTGCACAACCGCTGGCACAATACCCAGTTGG
$AP45^{D106N}$	AP45MutD106N-F	GTGATTTTTAACACTGGTAGCTCCAATTTGTG
	AP45MutD106N-R	TGTGAATTTCTGAGGAGGAGTCCCAACACC
$AP45^{D293N}$	AP45MutD293N-F	CAATTGCAAATTCTGGAACTTCCTTGTTGGC
	AP45MutD293N-R	CAGCACAACCACCAGCACAATACCCGGTTG
Primers used to identify transgenic plants		

GUS	GUS-F	GGGCGAACAGTTCCTGATTAACC
	GUS-R	CAGTACCTTCTCTGCCGTTTCCA
zCas	zCas-F	TGAGAACATCGTCATTGAGATGG
	zCas-R	TCAGCTTGTCATTCTCATCGTAC
Hyg	Hyg-F	GAGCTTGTCGATCGACAGAT
	Hyg-R	CATATGCGCGATTGCTGATC
Kana	Kana-F	ATGATTGAACAAGATGGATTGCACG
	Kana-R	GGCAGGAGCAAGGTGAGATGACA
Primers used for RT-qPCR		
AP17	RT-AP17-F	AGTGTTCCTTCCATGCCCACGG
	RT-AP17-R	TTAAGCTGCCTCTGCAAATCCAAC
AP45	RT-AP45-AT-F	GTTTGCTGTGGTATTATCCGAGTC
	RT-AP45-AT-R	CTGAGCATCAAAATAGTTCTTTAACG
Potri.001G062500/JAZ1	RT-0625-F	CAGTGCTGAACCAATGACTGATC
	RT-0625-R	TACCACCTATCTTGTAGCCATCC
Potri.002G176900/bHLH14	RT-1769-F	GCAGCTGATAGAATCCACATGG
	RT-1769-R	GTCATGCGGCTATTGCTGAAGG
Potri.013G157900/C4H1	RT-1579-F	CCATAGACCATATCCTCGATGC
	RT-1579-R	CTTGCTCTCAGCTGGAATGTCG
Potri.015G003100/COMT1	RT-0031-F	CTCTTGTCCCCATCCGATATAGC
	RT-0031-R	TAGAATGGTCAGACATTCCCTTG
Potri.001G024800/Annexin D3	RT-0248-F	ACAGCTGCAAGTGGTAGTTGAG
	RT-0248-R	TTCCACAACTCTTGATGTCCTGG
Potri.019G031200/Apyrase 2	RT-0312-F	CTTCGGCAGCATACAAGTTCAC
	RT-0312-R	GTTGAGCAATGGGACAAGTGAG
PtActin2	PtActin2-F	AACATGGGATTGTTAGCAACTGG
	PtActin2-R	TCCATCACCAGAATCCAGCACA
PteIF5A	PteIF5A-F	TCGTTCCTTCATCTCACAACTGT
	PteIF5A-R	AGACTCACAAAGCCATCTTCAGA
PtEF1β	PtEF1β-F	AACCTGGTCGTGATTTCCCT

	PtEF1β-R	ATCACCAGCAGCCTCCTTG
AtActin2	ATActin2-F	TCTTCTTCCGCTCTTTCTTTCC
	ATActin2-R	TCTTACAATTTCCCGCTCTGC
AtUBQ10	AtUBQ10-F	TAATCCCTGATGAATAAGTGTTCTAC
	AtUBQ10-R	AAAACGAAGCGATGATAAAGAAG
AtGAPDH	AtGAPDH-F	TTGGTGACAACAGGTCAAGCA
	AtGAPDH-R	AAACTTGTCGCTCAATGCAA

Table S2. The sequences of typical APs from different species.

>Cardosin A
MGTSIKANVLALFLFYLLSPTVFSVSDDGLIRIGLKKRKVDRIDQLRGRRALMEGNARKDFGFRGTVRDSGSAVVALTNDRD
TSYFGEIGIGTPPQKFTVIFDTGSSVLWVPSSKCINSKACRAHSMYESSDSSTYKENGTFGAIIYGTGSITGFFSQDSVTIGDLV
VKEQDFIEATDEADNVFLHRLFDGILGLSFQTISVPVWYNMLNQGLVKERRFSFWLNRNVDEEEGGELVFGGLDPNHFRGD
HTYVPVTYQYYWQFGIGDVLIGDKSTGFCAPGCQAFADSGTSLLSGPTAIVTQINHAIGANGVMNQQCKTVVSRYGRDIIEM
LRSKIQPDKICSHMKLCTFDGARDVSSIIESVVDKNNDKSSGGIHDEMCTFCEMAVVWMQNEIKQSETEDNIINYANELCEH
LSTSSEELQVDCNTLSSMPNVSFTIGGKKFGLTPEQYILKVGKGEATQCISGFTAMDATLLGPLWILGDVFMRPYHTVFDYGN
LLVGFAEAA

>Cardosin B
MGTPIKASLLALFLFFLLSPTAFSVSNGGLLRVGLKKRKVDRLDQLRAHGVHMLGNARKDFGFRRTLSDSGSGIVALTNDRD
TAYYGEIGIGTPPQNFAVIFDTGSSDLWVPSTKCDTSLACVIHPRYDSGDSSTYKGNGTTASIQYGTGAIVGFYSQDSVEVGDL
VVEHQDFIETTEEDDTVFLKSEFDGILGLGFQEISAGKAVPVWYNMVNQGLVEEAVFSFWLNRNVDEEEGGELVFGGVDPN
HFRGNHTYVPVTRKGYWQFEMGDVLIGDKSSGFCAGGCAAIADSGTSFFAGPTAIITQINQAIGAKGVLNQQCKTLVGQYG
KNMIQMLTSEVQPDKICSHMKLCTFDGAHDVRSMIESVVDKNNDKSSGGEICTFCEMALVRMQNEIKRNETEDNIINHVNE
VCDQLPTSSAESIVDCNGISSMPNIAFTIGSKLFEVTPEQYIYKVGEGEAATCISGFTALDIMSPQGPIWILGDMFMGPYHTVF
DYGKLRVGFAEAV

>OsAsp6
MGRNHLCLVTCLWILSCAVLLHASPDGLLRISLNKKRLDKKTLDGAKLAREESHRLRADGLGDDIVPLDNYLDTQYFGEIGI
GTPPQNFTVIFDTGSSNLWVPSVKCYFSIACYLHHRYKSKGSSSYKKNGESCSISYGSGSIAGFFSEDSVLVGDLAVKNQMFIE
TTREPSLTFIIGKFDGILGLGFPEISVGGAPPIWQGMKEQQLIEKDVFSFWLNRDPDAPTGGELIFGGVDPNHYKGSHTYVPVT
RKGYWQFEMGDLLIDDYSTGFCSGGCAAIADSGTSLLGGPTTIVAQINHAIGAEGIVSMECKQVVRDYGDMILEMLIAQASP
MKLCSQIGLCAFDGTRSVRNNIESVVDKEKVGSDLSCTACEMAVVWIQNQLRHNQTRELILQYADQLCERLPSPNGESAVD
CDEISNMPNLSFTIANKTFTLTPEQYVVKLEQQGQTVCISGFMAFDVPPPRGPLWILGDVFMAAYHTVFDFGKNRIGFAESA

>OsAsp9
MGHTASRICTLLSILFVSCMLASAANDGLIRIALKKRPIMESIYGELVPKSGTVDHEVALGYSGVRMNSADEGFYDPVTEAIN
HVRVHQQRMLRDIEAAAMEGRLKHFWSYRGFRERGSLKNGTQNHPLALKNFLNAQYFGEIGVGCPPQNFTVVFDTGSSNL
WVPSAKCVFSLACYFHRKYESRSSSTYMENGTPASIHYGTGSIHGYYSQDQVTIGDLVVNNQEFIEATHEPGLTFLAAKFDGI
LGLGFKEISVEGADPVWYNMIQQSLVTDKVFSFWLNRNANDINGGEIVFGGADESHYKGDHTYTRVTRKAYWQFEMGDFL
IGGRSTGICVDGCAVIADSGTSLIAGPIAAIAQIHAHIGATGVANEECKQVVARHGHEMLELLQDKTPPAQVCSKIGLCKSDG
AHGISDGIESVLGETHKSADEVSDATCNACEMAVTWMQSEFVQNHTKEGKLEYANQLCGNMPSPVGSYVDCRHIGHLPNV
AFSIGGRAFELTPEQQYILKFGEGFLAHCMSGFTALDIPPPIGPLWILGDVFMGAYHTIFDYGKMRVGFADSA

>OsAsp10
MGTRRVGLLLLLLLAAVLLQPLLAAAAAEGVVRIALKKRQVDETGRVGGHLAGEDAQRLLARRHGFLTNDAARAASRKA
RAEAEGDIVALKNYLNAQYYGEIAIGTPPQMFTVIFDTGSSNLWVPSSKCHLSIACYFHSRYKAGQSSTYKKNGKPASIHYGT
GAISGYFSQDSVKVGDVAVKNQDFIEATREPSITFMVAKFDGILGLGFKEISVGNAVPIWYNMVRQGLVVDPVFSFWFNRHA
DEGQGGEIVFGGIDPNHYKGNHTYVPVTRKGYWQFNMGDVLIGGNSTGFCAAGCAAIADSGTSLLTGPTAIITQINEKIGAT
GVVSQECKAVVSQYGQQILDQLRAETKPAKVCSSVGLCTFDGTHGVSAGIRSVVDDEVGKSSGPFSSAMCNACETAVVWM
HTQLAQNQTQDLVLQYIDQLCDRLPSPMGESSVDCSSLASMPDIAFTIGGNKFVLKPEQYILKVGEGTATQCISGFTAMDIPPP
RGPLWILGDVFMGAYHTVFDYGNLKVGFAEAA

>OsAsp41
MAKRHLLLVTTCLWALSCALLLHASSDGFLRVNLNKKRLDKEDLTAAKLAQQGNRLLKTGSSDSDPVPLVDYLNTQYYGVI
GLGSPPQNFTVIFDTGSSNLWVPSAKCYFSIACYLHSRYNSKKSSSYKADGETCKITYGSGAISGFFSKDNVLVGDLVVKNQK
FIEATRETSVTFIIGKFDGILGLGYPEISVGKAPPIWQSMQEQELLADDVFSFWLNRDPDASSGGELVFGGMDPKHYKGDHTY
VPVSRKGYWQFNMGDLLIDGHSTGFCAKGCAAIVDSGTSLLAGPTAIVAQVNHAIGAEGIISTECKEVVSEYGEMILNLLIAQ
TDPQKVCSQVGLCMFDGKRSVSNGIESVVDKENLGSDAMCSVCEMAVVWIENQLRENKTKELILNYANQLCERLPSPNGES

TVSCHQISKMPNLAFTIANKTFILTPEQYIVKLEQGGQTVCISGFMAFDIPPPRGPLWILGDVFMGAYHTVFDFGKDRIGFAKS
A
>OsAsp44
MGTRSVALVLLAAVLLQALLPASAAEGLVRIALKKRPIDENSRVAARLSGEEGARRLGLRGANSLGGGGGEGDIVALKNYM
NAQYFGEIGVGTPPQKFTVIFDTGSSNLWVPSAKCYFSIACFFHSRYKSGQSSTYQKNGKPAAIQYGTGSIAGFFSEDSVTVG
DLVVKDQEFIEATKEPGLTFMVAKFDGILGLGFQEISVGDAVPVWYKMVEQGLVSEPVFSFWFNRHSDEGEGGEIVFGGMDP
SHYKGNHTYVPVSQKGYWQFEMGDVLIGGKTTGFCASGCSAIADSGTSLLAGPTAIITEINEKIGATGVVSQECKTVVSQYG
QQILDLLLAETQPSKICSQVGLCTFDGKHGVSAGIKSVVDDEAGESNGLQSGPMCNACEMAVVWMQNQLAQNKTQDLILN
YINQLCDKLPSPMGESSVDCGSLASMPEISFTIGGKKFALKPEEYILKVGEGAAAQCISGFTAMDIPPPRGPLWILGDVFMGA
YHTVFDYGKMRVGFAKSA
>VvAP10
MRLKYILVANCLLWAWACCLALDDSSDGLVRIGLKKKPLDLARLHAARITRGNGFHAQGLGKVDDNYPKANTVYLKNYM
DAQYYGEIGIGSPPQTFSVVFDTGSSNLWVPSSKCYFSIACYFHARYRAVLSRTYSKNGRHCKINYGSGSISGFFSQDHVQIGE
IVIKNQVFTEATKEGLFAFSLAQFDGILGLGFQNASVGKIPPIWYNMVQQSLVSMEIVSFWLNRDPKAKIGGEVIFGGVDWR
HFMGDHTFVPITRKDYWQIEVGDILIAGSSTGFCEGGCAAIVDTGTSMIAGPTTVVTQINHAIGAEGIVSFNCKNVVNKYGR
LIWQFLVSGFQPENVCSDIGLCAYNGTKNASAGMETVIGNGDNAACTFCEMIAFWIQVQLKEHKAKEKVFQYVNELCENLP
NPGGKDFVNCDALATMPVISFAIGDKYFPLTAEQYTLKVEVNCTTVCLSGFTALDVPRPDGPLWVLGDVFLGAYHTIFDFGN
LQVGFAKSIL
>VvAP27
MDAQYFGEIGIGTPPQTFTVIFDTGSSNLWVPSSKCYFSVPCYFHSKYKSSQSSTYRKNGKSADIHYGTGAISGFFSEDNVKV
GDLVVKNQEFIEATREPSVTFLVAKFDGILGLGFQEISVGNAVPVWYNMVKQGLVKEPVFSFWLNRKTDDDEGGELVFGGV
DPDHFKGEHTYVPVTQKGYWQFDMGEVLIDGETTGYCAGGCAAIADSGTSLLAGPTAVVAMINHAIGATGVVSQECKTVV
AQYGETIMDLLLSEASPQKICSQIGLCTFDGTRGVGMGIESVVDEKNGDKSSGVHDAGCSACEMAVVWMQSQLRQNQTKE
RILEYVNELCDRLPSPMGESAVDCLQLSSMPNVSLTIGGKVFDLSANEYVLKVGEGAAAQCISGFIAMDVPPPRGPLWILGD
VFMGRYHTVFDYGNMRVGFAEAA
>VvAP34
MRQGVVWAAFCLWALICPLLPVYSHGSVRIGLKKRPLDFNNMRTARIAQMQGKIGGGVMSKYHGFDDPDGEFVSLKNYLD
AQYFGEIGIGTPPQNFTVVFDTGSSNLWVPSSKCYFSIACFFHNKYKARLSSTYTKIGRPGEIHYGSGSISGFFSQDNVEVGSL
VVKDQVFIEATREGSLTFALAKFDGIMGLGFQGISVGNATPVWSTMLQQGLLHEELFSFWLNRNPNANEGGEIVFGGVDKR
HFRGKHTFVPVTQAGYWQFRMGDFLISNQTTGVCEGGCSAIVDSGTSLIAGPTLVVTQINHAIGAEGIVSMECKEVVSQYGN
MMWDLLVSGVLPSKVCSQIGLCMASPGIRTVVEKEKMESVEEVGDVVFCNACEMIAVWIQSQLKQMKTKDKVLRYVTEL
CGSLPSPMGESVIDCTSVANMPNITFIIGDKAFDLTPDQYILRTGDGSATVCLSGFTALDVPPPKGPLWILGEIFMGVYHTVFDF
GDLRIGFAEAA
>VvAP35
MDAQYYGEIGIGTPPQNFTVVFDTGSANLWVPSTKCHFSIACLFHSKYNSRLSTTYIDLGKEGEIHYGSGSISGVFSQDNVQV
GSMAIKNQVFIEATREASLVFVLGKFDGILGLGFEEIVVGNATPVWYNLLRQGLVQEDIFSFWLNRDPQATDGGEIVFGGVD
KRHFKGQHTYASITQKGYWQFEMGEFLIGYQSTGFCEAGCAAIVDSGTSLIAGPTAIVTEINHAIGAEGIVSQECKEVVSQYG
NMIWDLLISRVQPDAVCSQIGLCNFNGSQIESPRIKTVVEEEDARGTKVGNEVWCTACEMTVIWIQNQLKQRKTKEIIFSYVT
ELCQSLPSPMGESVVDCGRVPYMPDVTFTIADKHFTLTPKEYVLKTGEGITTVCLSGFIALDVPPPRGPLWILGDIFMGVYHT
VFDYGNLQVGFAEAMQ
>VvAP45
MGTKCRTVAVALFLSILMFSPEFSASDGGLVRIGLKKRAFDQTNRLAARIESKQGEALGTSIRKYNLHGNAAGSKHTYVVAL
HNYMDAQYFGEISIGTPPQKFTVIFDTGSSNLWVPSSKCYFSVACYFHSKYKSSQSSTYKKNGTSADIHYGTGAISGFFSKDD
VKVGDLAVINQEFIEATKEPSITFALAKFDGILGLGFQEISVGNAVPVWYNMINQELIKEPIFSFWFNRNSNEEVGGEIVFGGID
SDHYKGKHTYVPVTKKGYWQFDLGDVMIGGKTTGFCASGCSAIADSGTSLLAGPTTIITEVNHAIGASGFVSQECRAVVQQ
YGQIIIDMLLTKEQPQKICSQIGLCAFNGIRGVSMGIESVVDENNSKASDGLHDTMCSACSMAVVWIQNKLGQNETIDRILKY

VNELCDRLPSPMGESAVDCGSLSSMPNVSLTIGGKVFDLSPKQYILKVGEGEIAQCISGFTALDVPPPHGPLWILGDVFMGQYHTVFDYGNMKVGFAEAA

>PtAP6

MGTILKPVAAATLFLCFLLLPMISSALSPPNDGLIRIGLKKRKYERNNRLAAKLESKEGESIKKYHLLRNLGGDAEDTDIVSLKNYMDAQYFGEIGIGTPPQKFTVIFDTGSSNLWVPSSKCYFSVACYFHSKYKSSHSRTYKENGKSAEIHYGTGAISGFFSQDHVKVGDLVVKNQEFIEATREPSVTFLVAKFDGILGLGFQEISVGKAVPVWYNMVEQGLVKEPVFSFWFNRNADEKEGGEIVFGGVDPDHYKGEHTYVPVTQKGYWQFDMGDVLIGGQTSGFCASGCAAIADSGTSLLAGPTTIITEVNHAIGATGVVSQECKAVVAQYGDTIMEMLLAKDQPQKICAQIGLCTFDGTRGVSMGIESVVNEHAQKASDGFHDAMCSTCEMAVVWMQNQLKQNQTQERILDYVNELCERLPSPMGESAVDCDGLSSMPNVSFTIGGRVFELSPEQYVLKVGEGDVAQCISGFTALDVPPPRGPLWILGDVFMGSFHTVFDYGNMRVGFAEAT

>PtAP11

MGTQFTGIWIALLLSFPVVLSARDDGLMRIGLKKKKLDHLGRRVVPGSVNFIPKEEGGGASKPAATKKYYNIGETEADIVALKNYLDAQYYGEITIGTPPQTFTVIFDTGSSNLWVPSSKCYFSLACYFHSKYKSSASTTYVKNGTSAAIQYGTGSISGFFSQDSVEVGDLVVKNQGFIEATKEPGVTFLASKFDGILGLGFQEISVGNAVPVWYNMVNQGLVKEKVFSFWLNRNVEGEEGGEIVFGGVDPNHYKGEHTYVPVTHKGYWQFDMGDLLIGTETTGLCAGGCKAIADSGTSLLAGPTTVITQINNAIGASGIVSEECKTVVAQYGKIILEMLVAQAQPRKVCSQISFCTFDGTQGVSMNIESVVEENSDKSSDGLHDAMCTACEMMVVWMENRLRLNDTEDQILDYVNNLCDRLPSPNGESAVECSSLSSMPSISFEIGGKLFELSPEQVWQFCSVYLLCSSYCIQNFSYFISFNLIKLEGSRLIARVDLSINSILLHSSPLKCRSIYLVEIFFHVLFHIQI

>PtAP17

MGVNLKAIVGFVFLSFLLFAVVSSASNDGLLRIGLKKVKLDKNNRIAARLDSKETLRASIRKYNLCGNLGESEDTDIVALKNYLDSQYYGEIGVGSPPQKFTVIFDTGSSNLWVPSSKCYLSVACYFHSKYDSGKSSTYKKNGKSAEIRYGSGSISGFFSNDAVEVGGLVVKDQEFIEATKEPNITFLVAKFDGILGLGFKEISVGDAVPVWDNMIKHGLIKEPVFSFWLNRNAEDEEGGEIVFGGMDPNHYKGKHTFVPVTRKGYWQFNMGDVHIGDKPTGYCASGCAAIADSGTSLLAGPTTIITMINQAIGASGVVSQQCKAVVSQYGEAIMDLLLSQAQPKRICSQIGLCTFDGTRGISIGIQSVVDEGNDKSSGVLGDAMCPACEMAVVWMRSQLKQNQTQDRILDYVNQLCERMPNPMGESAVDCESVPSMPTVAFTIGGKEFELAPEEYILKVGQGSAAQCISGFTALDIPPPRGPLWILGDIFMGRYHTVFDSGKLRVGFAEAA

>PtAP19

MLNTWEKLVLARPHKTSVIFDTGSSNLWVPSSKCYFSIACYFHSKYKSSLSSTYIKNGNSCEIHYGSGSISGFLSQDNVQVGGLVVKDQVFIEVTKEGSLSFVLGKFDGILGLGFQEISVGNVVPVWYNMVQQDLVDDEVFSFWLNRNPEAKEGGELVFGGVDPKHFKGKHTYVPVTKKGYWQINMGDFLIGNHSTGLCEEGCAAIVDSGTSLLAGPTPIITEINHAIGAEGVVSAECKEVVSQYGDLIWELLISGVKPNKVCAQLGLCIFNGDEYVSTGIESVVEKENKEGSSAGDDLLCIACEMLVIWVQNQLREKETKEAAINYLDKLCESLPSPMGESVIDCNSISTMPNISFTIGDKPFSLTPEQYVLKTGEGIAQVCISGFMALDVPPPRGPLWILGDVFMGVYHTVFDYGNLEVGFAEAA

>PtAP42

MESIRFLLISICMGAWLGGSLSSDGLARVGLKKRNLDLNSIHAARITRPQATSFARVTSNAEIVYLKNYLDTQYYGEIGIGSPPQIFTVVFDTGSSNLWVPSSKCLLSITCYFHSKFIARLSRTYTKIGIPCKIQYGSGSVSGFLSQDHVKVGDDIIINQEFAEVTREGFLALLGVQFDGILGLAFQDIAVAKATPVWYNMAEQGHVSQKVFSLWLNRNPSSELGGEVVFGGLDWRHFKGDHTYVPVTGRGYWQIQVGDIFIANNSTGLCAGGCSAIVDSGTSFLSGPTRIVAQINHAIGARGIVSLECKEVVSKYWNSIWDSMISGLRPEIICVDVGLCLYNNNTVIETVVDGEATDRLSVDEGGALCTFCEMIVFWIQVQLKEKKAKEKIFHYVDELCERLPNPLGKSFINCDEITAMPYVSFTIGNRSFPLSPEQYIVRVEESYATICLSGFAALDMPPRQGPLWILGDVFLGAYHTVFDFGNHRIGFAKAA

>PtAP45

MGVNLKAIGGFVLLSFLLFAVVLSESNDGLLRIGLKKVKFDKNNRIAARLDSQEALRASIRKYNLLGNLGESEDTDIVALKNYFDAQYYGEIGVGTPPQKFTVIFDTGSSNLWVPSSKCYLSVACYFHSKYNSGKSSSYKKNGKSAEIQYGSGSISGFFSIDAVEVGNLVVKDQEFIEATKEPSITFLVGKFDGILGLGFKEIAVGGAVPVWDNMIKQGLIKEPVFSFWLNRNADDEEGGEIVFGGMDPNHYKGKHTYVPVTQKGYWQFDMGDVIVGDKSTGYCAGGCAAIADSGTSLLAGPTAIITMINHAIGASGVVSQQCKAVVSQYGEVIMDLLLSEVQPKKICSQIGLCTFDGTRGISMGIQSVVDEGNDKSSGVLGD

>PtAP47

MSALDFYTLFRLNSILHPRMGNKILLKAFCLWALTCFLLPASSNGLVRIGLKKRHLDLQTIKDARIARQEGKAGVGASSRVHD
LGSSDGDIIPLKNYLDAQYLGEIGIGSPPQNFTVVFDTGSSNLWVPSSKCYFSIACYFHSKYKSSRSSTYTKNGNFCEIHYGSG
SVSGFFSQDNVQVGDLVVKDQVFVEATKEGSLSFILGKFDGILGLGFQEISVGNVVPLWYNMIQQDLVDDEVFSFWLNRNPE
AKEGGELVFGGVDPKHFKGKHTYVPVTQKGYWQINMGDFLIGKHSTGLCEGGCAAIVDSGTSLLAGPTPIITEINHAIGAEG
LVSAECKEVVSHYGDLIWELIISGVQPSKVCTQLGLCIFNEAKSARTGIESVVEKENKEKSSAGNDLPCTACQMLVIWVQNQ
LREKATKETAINYLDKLCESLPSPMGQSSIDCNSISTMPNITFTIGDKPFSLTPEQYILKTGEGIAQVCISGFMALDVPPPRGPLW
ILGDVFMGAYHTIFDYGNLEVGFAEAA

>AT1G11910

QYYGEIAIGTPPQKFTVVFDTGSSNLWVPSSKCYFSLACLLHPKYKSSRSSTYEKNGKAAAIHYGTGAIAGFFSNDAVTVGDL
VVKDQEFIEATKEPGITFVVAKFDGILGLGFQEISVGKAAPVWYNMLKQGLIKEPVFSFWLNRNADEEEGGELVFGGVDPNH
FKGKHTYVPVTQKGYWQFDMGDVLIGGAPTGFCESGCSAIADSGTSLLAGPTTIITMINHAIGAAGVVSQQCKTVVDQYGQ
TILDLLLSETQPKKICSQIGLCTFDGTRGVSMGIESVVDKENAKLSNGVGDAACSACEMAVVWIQSQLRQNMTQERILNYV
NELCERLPSPMGESAVDCAQLSTMPTVSLTIGGKVFDLAPEEYVLKVGEGPVAQCISGFIALDVAPPRGPLWILGDVFMGKY
HTVFDFGNEQVGFAEA

>AT1G62290

QYYGEIAIGTPPQKFTVIFDTGSSNLWVPSGKCFFSLSCYFHAKYKSSRSSTYKKSGKRAAIHYGSGSISGFFSYDAVTVGDLV
VKDQEFIETTSEPGLTFLVAKFDGLLGLGFQEIAVGNATPVWYNMLKQGLIKRPVFSFWLNRDPKSEEGGEIVFGGVDPKHFR
GEHTFVPVTQRGYWQFDMGEVLIAGESTGYCGSGCSAIADSGTSLLAGPTAVVAMINKAIGASGVVSQQCKTVVDQYGQTI
LDLLLAETQPKKICSQIGLCAYDGTHGVSMGIESVVDKENTRSSSGLRDAGCPACEMAVVWIQSQLRQNMTQERIVNYINEI
CERMPSPNGESAVDCSQLSKMPTVSFTIGGKVFDLAPEEYVLKIGEGPVAQCISGFTALDIPPPRGPLWILGDVFMGKYHTVF
DFGNEQVGFAEA

>AT1G69100

VFYGEISVGSPPQKFNVVFDTGSTDLWVPSKEWPEETDHKHPKFDKDASKTCRLMKGGEVNIAYETGSVVGILAQDNVNV
GGVVIKSQDLFLARNPDTYFRSVKFDGVIGLGIKSSRAQGSVTVWENMVKQKLITKPIFSLYLRPHKGDGGEDPNGGQIMFG
GFDPKQFKGEHVYVPMKLSDDRWKIKMSKIYINGKPAINFCDDVECTAMVDSGSTDIFGPDEAVGKIYKEIGATKVIIRCEQF
PALPDIYFEIGGKHLRLTKHDYVEVKTNPKKRCRLRIVKSKNRRKDWVLGEAFMTKFHTVFDYGDVKTPRIGFAEA

>PASPA3

QYYGDITIGTPPQKFTVIFDTGSSNLWIPSTKCYLSVACYFHSKYKASQSSSYRKNGKPASIRYGTGAISGYFSNDDVKVGDIV
VKEQEFIEATSEPGITFLLAKFDGILGLGFKEISVGNSTPVWYNMVEKGLVKEPIFSFWLNRNPKDPEGGEIVFGGVDPKHFK
GEHTFVPVTHKGYWQFDMGDLQIAGKPTGYCAKGCSAIADSGTSLLTGPSTVITMINHAIGAQGIVSRECKAVVDQYGKTM
LNSLLAQEDPKKVCSQIGVCAYDGTQSVSMGIQSVVDDGTSGLLNQAMCSACEMAAVWMESELTQNQTQERILAYAAELC
DHIPTQNQQSAVDCGRVSSMPIVTFSIGGRSFDLTPQDYIFKIGEGVESQCTSGFTAMDIAPPRGPLWILGDIFMGPYHTVFDY
GKGRVGFAKA

>AT4G22050

LYYGKIQIGNPGQTFTVLFDTGSSSLWVPSENWLAKTENPRNRYISSASRTFKENGTKAELKYGKGSLTGFLSVDTVTVGGIS
ITSQTFIEGVKTPYKEFFKKMPFDGILGLRFTDPLNFGTSVWHSMVFQGKIAKNVFSIWLRRFSNSGEINGGEVVFGGIIPAHF
SGDHTYVDVEGPGNFFAMSNIWVGGKNTNICSSGCKAIVDSGSSNINVPMDSADEIHRYIGVEPNCNNFETLPDVTFTIGGK
AFVLTPLDYIRRSRSQCTSKFVGKTNRSHWTLGIPFMRVFHTVFDYQNTLAVKVGFAKS

Table S3. ***AP17*** **and/or** ***AP45*** **mutations by the Cas9/gRNA in multiple transgenic lines.**

Gene: Potri.004G007600 (*AP17*)

Vector: Cas9/gRNA-AP17-1
gRNA: gAP17-a (ATTAGGAAGTATAATCTTTGCGG); gAP17-b (CCTCAGAAGTTCACTGTGATCTT)
Target sequences: AP17-a, ATTAGGAAGTATAATCTTTGCGG; AP17-a, CCTCAGAAGTTCACTGTGATCTT

**Line (#)**	**gAP17-a**<br>ATTAGGAAGTATAATCTTTGCGG	**Mutation types**<br>(Number of plasmids containing the cloned PCR products)		**gAP17-b**<br>CCTCAGAAGTTCACTGTGATCTT	**Mutation types**<br>(Number of plasmids containing the cloned PCR products)	
ap17-1#	ATTAGGAAGTATAATCTTATGCGG<br>ATTAGGAAGTATAATCTTTG.GG<br>ATTAGGAAGTATAATCTCATTTGCGG	+1 (×12)<br>-1 (×8)<br>+3 (×10)	chimera	CCTCAGCAAGTTGCACTGTGATCTT<br>CCTCAGAAGTCGTCACTGTGATCTT<br>CCCATCAGCAAGTTCACTGTGATCTT	+2 (×12)<br>+2 (×8)<br>+3 (×10)	chimera
ap17-2#	ATTAGGAAGTAT......TGCGG<br>ATTAGGAAGTATAATCTT.GCGG	-6 (×16)<br>-1 (×14)	biallelic	CCTCAG..GTTCACTGTGATCTT<br>CCTCAGA.GTTCACTGTGATCTT	-2 (×16)<br>-1 (×14)	biallelic
ap17-3#	ATTAGGAAGTATAATCTTTTGCGG<br>ATTAGGAAGTATAAT...TGCGG	+1 (×15)<br>-3 (×15)	biallelic	CCTCAGA.GTTCACTGTGATCTT<br>CCTCAG....TCACTGTGATCTT	-1 (×15)<br>-4 (×15)	biallelic
ap17-4#	ATTAGGAAGTATAATCTTTGCGG<br>ATTAGGAAGTATAATCTTTTGCGG	0 (×18)<br>+1 (×12)	heterozygous	CCTCAGA.GTTCACTGTGATCTT	-1 (×30)	homozygous
ap17-5#	ATTAGGAAGTATAATCTTATGCGG<br>...........................	+1 (×18)<br>-343 (×12)	biallelic	CCTCAGAAGTTCACTGTGATCTT<br>...........................	0 (×18)<br>-343 (×12)	heterozygous

Vector: Cas9/gRNA-AP17-2
gRNA: gAP17-c (GTATCATCTGCGTCGAATGATGG); gAP17-d (TGTTGCATTGAAGAATTATCTGG)
Target sequences: AP17-c, GTATCATCTGCGTCGAATGATGG; AP17-d, TGTTGCATTGAAGAATTATCTGG

**Line (#)**	**gAP17-c**<br>GTATCATCTGCGTCGAATGATGG	**Mutation types**<br>(Number of plasmids containing the cloned PCR products)		**gAP17-d**<br>TGTTGCATTGAAGAATTATCTGG	**Mutation types**<br>(Number of plasmids containing the cloned PCR products)	
ap17-6#	GTATCATCTGCGT....TGATGG<br>GTATCATCTGCGTC...TGATGG	-4 (×13)<br>-3 (×17)	biallelic	TGTTGCATTGAA......TCTGG<br>TGTTGCATTGAAGAAT.ATCTGG	-6 (×13)<br>-1 (×17)	biallelic
ap17-7#	GTATCATCTGCGTCGAA.GATGG<br>GTATCATCTGCGTCGAAATGATGG	-1 (×14)<br>+1 (×16)	biallelic	TGTTGCATTGAAGAATTATCTGG<br>TGTTGCATTGAAGAA..ATCTGG<br>TGTTGCATTGAAGAAT.ATCTGG	0 (×8)<br>-2 (×12)<br>-1 (×10)	chimera
ap17-8#	GTATCATCTGCGTCGAAT........<br>GTATCATCTGCGTCGAAATGATGG	-17 (×15)<br>+1 (×15)	biallelic	.........................GG<br>TGTTGCATTGAA.....ATCTGG	-45 (×15)<br>-5 (×15)	biallelic
ap17-9#	GTATCATCTGCGTCGAATGATGG<br>GTATCATCTGCGTCGAAATGATGG	0 (×11)<br>+1 (×19)	heterozygous	TGTTGCATTGAA.....ATCTGG<br>TGTTGCATTGAAGAAT...CTGG	-5 (×11)<br>-3 (×19)	biallelic
ap17-10#	GTATCATCTGCGTC...TGATGG	-3 (×30)	homozygous	TGTTGCATTGAA......TCTGG	-6 (×30)	homozygous
ap17-11#	GTATCATCTGCGTCGAA.GATGG<br>GTATCATCTGCGTCGAAATGATGG	-1 (×12)<br>+1 (×18)	biallelic	TGTTGCATTGAAGAAT...CTGG<br>TGTTGCATTGAAGAAT.ATCTGG	-3 (×12)<br>-1 (×18)	biallelic

ap17-12#	GTATCATCTGCGTCGAATGATGG<br>GTATCATCTGCGTC...TGATGG	0 (×15)<br>-3 (×15)	heterozygous	TGTTGCATTGAAGAATTATCTGG<br>TGTTGCATTGAA......TCTGG<br>TGTTGCATTGAAGAAT.ATCTGG	0 (×14)<br>-6 (×12)<br>-1 (×4)	chimera
**Gene**: Potri.011G007600 (*AP45*)						
**Vector**: Cas9/gRNA-AP45-1<br>**gRNA**: gAP45-e (GTGAACTTGAAAGCAATTGGGGG); gAP45-f (ACACTGGTAGCTCCAATTTGTGG)<br>**Target sequences**: AP45-e, GTGAACTTGAAAGCAATTGGGGG; AP45-f, ACACTGGTAGCTCCAATTTGTGG						
**Line (#)**	**gAP45-e**<br>GTGAACTTGAAAGCAATTGGGGG	**Mutation types**<br>(Number of plasmids containing the cloned PCR products)		**gAP45-f**<br>GTCACAGTGATTTTTGACACTGG	**Mutation types**<br>(Number of plasmids containing the cloned PCR products)	
ap45-1#	GTGAACTTGAAAGCAATTGTGGGG<br>............................	+1 (×14)<br>-331 (×16)	biallelic	............................<br>............................	-303 (×14)<br>-331 (×16)	biallelic
ap45-2#	GTGAACTTGAAAGCAAT......	-312 (×30)	homozygous	....	-312 (×30)	homozygous
ap45-3#	GTGAACTTGAAAGCAC.......	-329 (×30)	homozygous	....	-329 (×30)	homozygous
ap45-4#	GTGAACTTGAAAGC..........<br>GTGAACTTGAAAGCAATT.....	-313 (×18)<br>-310 (×12)	biallelic	............................<br>............................	-313 (×18)<br>-310 (×12)	biallelic
ap45-5#	GTGAACTTGAAAGCA........<br>GTGAACTTGAAAGCAATT.....	-329 (×17)<br>-310 (×13)	biallelic	............................<br>............................	-329 (×17)<br>-310 (×13)	biallelic
ap45-6#	GTGAACTTGAAAGCAATT.....<br>............................	-310 (×16)<br>-347 (×14)	biallelic	............................<br>............................	-310 (×16)<br>-347 (×14)	biallelic
ap45-7#	GTGAACTTGAAAGCAATT.....<br>GTGAACTTGAAAGCAATT.....	-375 (×17)<br>-310 (×13)	biallelic	............................<br>............................	-375 (×17)<br>-310 (×13)	biallelic
**Gene**: Potri.004G007600 (*AP17*)/Potri.011G007600 (*AP45*)						
**Vector**: Cas9/gRNA-AP17/45-1<br>**gRNA**: gAP17/45-g (CCAAATCATTACAAGGGCAAGCA); gAP17/45-h (TCTGGAACTTCCTTGTTGGCAGG)<br>**Target sequences**: AP17-g, CCAAATCATTACAAGGGCAAGCA; AP17-h, TCTGGAACTTCCTTGTTGGCAGG<br>AP45-g, CCAAATCATTACAAGGGCAAGCA; AP45-h, TCTGGAACTTCCTTGTTGGCAGG						
**Line (#)**	**gAP17/45-g**<br>AP17, CCAAATCATTACAAGGGCAAGCA<br>AP45, CCAAATCATTACAAGGGCAAGCA	**Mutation types**<br>(Number of plasmids containing the cloned PCR products)		**gAP17/45-h**<br>AP17, TCTGGAACTTCCTTGTTGGCAGG<br>AP45, TCTGGAACTTCCTTGTTGGCAGG	**Mutation types**<br>(Number of plasmids containing the cloned PCR products)	
ap17ap45-1# -AP17	CCAAATCATTACAAGGGCAAGCA	0 (×30)	WT	TCTGGAACTTCCTTGTTTGGCAGG<br>TCTGGAACTTCCTTGTTCGGCAGG	+1 (×17)<br>+1 (×13)	biallelic
-AP45	CCAAATCATTACAAGGGCAAGCA	0 (×30)	WT	TCTGGAACTTCCTTGTTTGGCAGG<br>TCTGGAACTTCCTTGTTAGGCAGG<br>TCTGGAACTTCCTTGTTGGCAGG	+1 (×19)<br>+1 (×6)<br>0 (×5)	chimera
ap17ap45-2# -AP17	CCAAATCATTACAAGGGCAAGCA	0 (×30)	WT	TCTGGAACTTCCTTGTTTGGCAGG	+1 (×30)	homozygous
-AP45	CCAAATCATTACAAGGGCAAGCA	0 (×30)	WT	TCTGGAACTTCCTTGTTTGGCAGG<br>TCTGGAACTTCCTTGT.GGCAGG	+1 (×14)<br>-1 (×10)	chimera

					TCTGGAACTTCCTTGTTGGCAGG<br>TCTGGAACTTCCTTGTTGGGCAGG	0 (×3)<br>+1 (×3)	
ap17ap45-3#	-AP17	CCAAATCATTACAAGGGCAAGCA	0 (×30)	WT	TCTGGAACTTCCTTGTTAGGCAGG<br>TCTGGAACTTCCTTGTTTGGCAGG	+1 (×13)<br>+1 (×17)	biallelic
	-AP45	CCAAATCATTACAAGGGCAAGCA	0 (×30)	WT	TCTGGAACTTCCTTGTTTGGCAGG	+1 (×30)	homozygous
ap17ap45-4#	-AP17	CCAAATCATTACAAGGGCAAGCA	0 (×30)	WT	TCTGGAACTTCCTTGTTTGGCAGG	+1 (×30)	homozygous
	-AP45	CCAAATCATTACAAGGGCAAGCA	0 (×30)	WT	TCTGGAACTTCCTTGTTTGGCAGG	+1 (×30)	homozygous
ap17ap45-5#	-AP17	CCAAATCATTACAAGGGCAAGCA	0 (×30)	WT	TCTGGAACTTCCTTGTTGGCAGG<br>TCTGGAACTTCCTTGTTTGGCAGG	0 (×18)<br>+1 (×12)	heterozygous
	-AP45	CCAAATCATTACAAGGGCAAGCA	0 (×30)	WT	TCTGGAACTTCCTTGTTGGCAGG	0 (×30)	WT
ap17ap45-6#	-AP17	CCAAATCATTACAAGGGCAAGCA	0 (×30)	WT	TCTGGAACTTCCTTGTTTGGCAGG	+1 (×30)	homozygous
	-AP45	CCAAATCATTACAAGGGCAAGCA<br>CCAAATCGGATTGACAAGGGCAAGCA	0 (×11)<br>+3 (×19)	heterozygous	TCTGGAACTTCCTTGTTTGGCAGG<br>TCTGGAACTTCCTTGTTGGCAGG	+1 (×11)<br>0 (×19)	heterozygous
ap17ap45-7#	-AP17	CCAAATCATTACAAGGGCAAGCA	0 (×30)	WT	TCTGGAACTTCCTTGTTTGGCAGG	+1 (×30)	homozygous
	-AP45	CCAAATCATTACAAGGGCAAGCA	0 (×30)	WT	TCTGGAACTTCCTTGTTGGCAGG	0 (×30)	WT
ap17ap45-8#	-AP17	CCAAATCATTACAAGGGCAAGCA	0 (×30)	WT	TCTGGAACTTCCTTGTTTGGCAGG	+1 (×30)	homozygous
	-AP45	CCAAATCATTACAAGGGCAAGCA	0 (×30)	WT	TCTGGAACTTCCTTGTTGGCAGG	0 (×30)	WT
ap17ap45-9#	-AP17	CCAAATCATTACAAGGGCAAGCA	0 (×30)	WT	TCTGGAACTTCCTTGTTTGGCAGG<br>TCTGGAACTTCCTTGT.GGCAGG	+1 (×14)<br>-1 (×16)	biallelic
	-AP45	CCAAATCATTACAAGGGCAAGCA	0 (×30)	WT	TCTGGAACTTCCTTGTTTGGCAGG	+1 (×30)	homozygous
ap17ap45-10#	-AP17	CCAAATTCATTACAAGGGCAAGCA<br>CCAAATCATTACAAGGGCAAGCA<br>CCAAAT..TTACAAGGGCAAGCA	+1 (×10)<br>0 (×12)<br>-2 (×8)	chimera	TCTGGAACTTCCTTGTTTGGCAGG<br>TCTGGAACTTCCTTGTTCGGCAGG	+1 (×17)<br>+1 (×13)	biallelic
	-AP45	CCAAATCATTACAAGGGCAAGCA	0 (×30)	WT	TCTGGAACTTCCTTGTTGGCAGG	0 (×30)	WT

Biallelic, two alleles of the gene were edited and the different mutations were induced. Chimeric, more than three different allelic mutations in a target editing site of the gene.

Table S4. The DEGs of developing xylem in the *ap17ap45* mutants compared with wild-type plants.

Gene	Log2 Fold Change	Up/Down	*P* Value	FDR	Description
Potri.004G007600	-3.885554695	Down	6.70E-76	1.43E-71	AP17
Potri.011G007600	-3.41804821	Down	4.88E-50	5.22E-46	AP45
Potri.005G107300	-1.735822038	Down	5.76E-06	0.002240084	Ganglioside induced differentiation-associated protein 2 (GDAP2)
Potri.004G235500	-1.686030296	Down	6.19E-05	0.010668852	(+)-Neomenthol dehydrogenase
Potri.019G093300	-1.675198356	Down	0.000226096	0.026123288	FLA30
Potri.010G011700	-1.552337347	Down	4.26E-05	0.008436991	Similar to senescence-associated protein SAG102
Potri.001G078800	-1.519869555	Down	0.000198188	0.0241111	MLO-like protein 13
Potri.011G167000	-1.397718504	Down	2.76E-06	0.001374334	Probable amino acid permease 7
Potri.001G093600	-1.363010758	Down	8.04E-08	0.000101106	Probable GABA transporter 2
Potri.003G133400	-1.362672123	Down	2.12E-05	0.005208568	Late embryogenesis abundant protein (LEA_2)
Potri.009G164900	-1.35333004	Down	1.46E-05	0.004027597	Alpha/beta hydrolase fold containing protein
Potri.017G143820	-1.307862753	Down	0.000534203	0.041673683	Apoptotic ATPase
Potri.004G039700	-1.299585271	Down	5.20E-06	0.002096316	Soluble epoxide hydrolase
Potri.007G069600	-1.294589843	Down	1.03E-06	0.00064831	Glutamine synthetase
Potri.007G124300	-1.277868355	Down	0.000125116	0.017594478	SANT Associated
Potri.016G082900	-1.2554558	Down	4.37E-07	0.000321957	SAP domain-containing ribonucleoprotein
Potri.009G060600	-1.248924063	Down	5.45E-05	0.00962711	Transcription factor NF-Y alpha
Potri.005G211000	-1.240643864	Down	4.47E-05	0.008454192	Calmodulin binding protein-like
Potri.009G063300	-1.206332802	Down	0.00011389	0.016403951	CAD9
Potri.013G157900	-1.203930724	Down	3.99E-06	0.001778461	C4H1
Potri.005G141600	-1.190587744	Down	6.73E-06	0.002480339	Cathepsin L
Potri.016G047800	-1.172260089	Down	1.58E-06	0.000962074	Cotton fibre expressed protein (DUF761)
Potri.017G130700	-1.161052312	Down	0.00032658	0.030887867	15.7 kDa heat shock protein, peroxisomal
Potri.017G063500	-1.15720705	Down	9.05E-05	0.014013176	Glycerophosphoryl diester phosphodiesterase
Potri.002G173400	-1.136555059	Down	9.83E-08	0.000116767	Kinesin light chain
Potri.006G258600	-1.108432533	Down	1.62E-05	0.004383777	Serine-rich protein-related
Potri.003G121500	-1.103693954	Down	1.97E-05	0.004942588	BCL2-associated athanogene (BAG)
Potri.014G095800	-1.10209037	Down	5.97E-07	0.00042546	Protein trichome birefringence-like 25

Potri.015G003100	-1.097799495	Down	4.74E-05	0.008813944	COMT1
Potri.007G134500	-1.088837871	Down	4.44E-05	0.008454192	MYB54
Potri.004G199300	-1.084402969	Down	4.86E-05	0.008888984	F-box protein
Potri.008G033900	-1.082696444	Down	2.30E-07	0.000196832	Threonine-specific protein kinase
Potri.012G055700	-1.072207643	Down	1.94E-07	0.000185168	BEE 3
Potri.017G074400	-1.069402503	Down	2.08E-07	0.000185168	Threonine-specific protein kinase
Potri.002G157000	-1.049849068	Down	3.98E-05	0.008180691	(+)-Neomenthol dehydrogenase
Potri.018G130100	-1.045135767	Down	2.53E-05	0.005986966	Tetratricopeptide repeat-containing domain
Potri.002G187300	-1.039888357	Down	0.000291682	0.03024419	Diglyceride acyltransferase
Potri.008G068700	-1.034598687	Down	0.000304824	0.030268867	Serine/threonine-protein kinase-like protein CCR3
Potri.002G016700	-1.032383529	Down	0.000406661	0.035657381	Probable prolyl 4-hydroxylase
Potri.007G121100	-1.018773806	Down	9.28E-06	0.003100478	B-box-type zinc finger
Potri.006G006000	-1.005258537	Down	1.92E-06	0.001023695	Oligopeptide transporter 3
Potri.006G018000	-1.003370975	Down	6.60E-07	0.000441123	Microtubule-associated protein 70-5
Potri.012G142600	1.002052613	Up	3.11E-06	0.001445658	60S ribosomal protein L36e
Potri.005G092500	1.016726128	Up	4.82E-06	0.002016521	60S ribosomal protein L44e
Potri.019G034900	1.034296997	Up	0.000639865	0.046703159	Putative H/ACA ribonucleoprotein complex subunit 1
Potri.005G176100	1.038321829	Up	0.000520977	0.041243974	EamA-like transporter
Potri.001G388801	1.039525129	Up	0.000376267	0.033651483	Polyphenol oxidase
Potri.002G088600	1.039715904	Up	4.46E-05	0.008454192	Nitrate reductase
Potri.015G087700	1.041947497	Up	4.91E-05	0.008888984	Histone acetyltransferase
Potri.002G130700	1.045965764	Up	0.000170762	0.02212144	Allene Oxide Synthase
Potri.011G154200	1.049118762	Up	8.17E-05	0.013024435	Alpha-glucosidase-like
Potri.001G389200	1.05282449	Up	9.00E-05	0.014013176	UDP-glycosyltransferase 74E2
Potri.007G045100	1.059270535	Up	3.52E-05	0.007449795	Subtilisin-like protease SBT1.9
Potri.002G186400	1.079112261	Up	0.000181809	0.022995096	IAA20.1
Potri.006G236500	1.08302306	Up	0.000207153	0.024599462	NADP-dependent malic enzyme 2
Potri.006G065500	1.083789011	Up	1.71E-06	0.0009819	Hydrophobic seed protein
Potri.018G087400	1.088964756	Up	0.000640188	0.046703159	Pre-mRNA processing protein PRP39
Potri.008G070950	1.108170563	Up	7.71E-08	0.000101106	Ribosomal protein L41

Potri.003G150800	1.109581871	Up	0.00015705	0.020594795	ERF5
Potri.015G096200	1.112231335	Up	3.09E-05	0.006682049	Programmed cell death protein 2 (PCDC2)
Potri.019G066100	1.145495691	Up	7.70E-05	0.012561387	Development/cell death domain protein
Potri.011G052600	1.158956044	Up	3.98E-06	0.001778461	Ubiquitin-conjugating enzyme
Potri.002G118800	1.182107895	Up	0.000528918	0.041511554	ARM repeat superfamily protein
Potri.001G167700	1.182279941	Up	0.000691284	0.048264399	Lipoxidase
Potri.018G014800	1.187184612	Up	1.14E-05	0.003537631	AP62
Potri.010G072200	1.208702178	Up	0.000329792	0.030917968	Extensin-like protein repeat
Potri.002G080000	1.229475469	Up	0.000246805	0.027193048	G2/mitotic-specific cyclin-B
Potri.007G092500	1.261532531	Up	4.91E-06	0.002016521	Annexin D1
Potri.009G067500	1.267980494	Up	0.000153973	0.020315907	Pre-mRNA branch site protein p14
Potri.003G072800	1.296277097	Up	1.64E-10	4.38E-07	Pectinesterase
Potri.018G140500	1.308420736	Up	8.41E-07	0.000544617	Uncharacterized protein
Potri.001G219300	1.308940514	Up	1.88E-10	4.46E-07	L-ascorbate oxidase
Potri.007G044500	1.310598125	Up	1.02E-05	0.003247496	Triglyceride lipase
Potri.015G075800	1.319911164	Up	2.85E-05	0.006474486	MYB-like DNA binding protein
Potri.012G032900	1.362497969	Up	0.000241513	0.026832422	ERF86
Potri.005G195000	1.383492092	Up	0.000465296	0.0388504	ERF76
Potri.010G150500	1.390530364	Up	2.42E-07	0.000199303	Stress responsive A/B Barrel Domain
Potri.003G141800	1.40566006	Up	0.000716425	0.049081979	Glutaredoxin-related protein
Potri.007G120002	1.415199417	Up	0.000496979	0.040086502	SRF-type transcription factor
Potri.009G112553	1.438544206	Up	3.63E-11	1.11E-07	Probable lipid transfer (LTP_2)
Potri.008G128400	1.484320135	Up	3.98E-10	8.51E-07	Copper Transport Protein Atox1
Potri.019G124750	1.509947989	Up	0.000371137	0.033472835	Proteinase inhibitor I3
Potri.008G220600	1.539236135	Up	0.000102571	0.015120368	SWEET17
Potri.006G242100	1.550405019	Up	2.03E-07	0.000185168	ATP-dependent protease cereblon
Potri.016G117500	1.55074021	Up	1.36E-05	0.00386606	Delta(12)-fatty acid dehydrogenase
Potri.019G082866	1.553245701	Up	0.000318147	0.030426544	Uncharacterized protein
Potri.014G100800	1.561731022	Up	0.000194082	0.023888669	MYB-like DNA binding protein
Potri.001G376200	1.573002065	Up	5.70E-05	0.009993026	Hypothetical protein

Potri.001G288600	1.594020448	Up	0.000666029	0.047613248	Pathogenesis-related protein 1
Potri.005G158800	1.600483323	Up	0.000567661	0.04353886	Zinc-finger homeodomain protein 1
Potri.004G038000	1.68508995	Up	0.000474131	0.038979066	AtNAC2-related
Potri.004G185300	1.685975787	Up	0.000530183	0.041511554	Cytochrome P450 94C1
Potri.001G191000	1.707382165	Up	3.03E-05	0.006677274	Acid phosphatase B
Potri.002G233000	1.714407613	Up	7.19E-05	0.012199975	Uncharacterized protein
Potri.016G137400	1.77178076	Up	2.19E-05	0.005330603	Costunolide synthase
Potri.002G031700	1.877803097	Up	4.34E-06	0.001854378	Translation initiation factor 1A
Potri.003G131300	1.878941366	Up	2.55E-05	0.005986966	Homeobox protein BEL1 homolog
Potri.001G387900	1.881493855	Up	1.61E-07	0.000172114	Polyphenol oxidase
Potri.019G079600	1.889649645	Up	0.000279975	0.029922281	Oligopeptide transporter
Potri.002G176900	1.929240628	Up	2.72E-08	4.85E-05	Transcription Factor BHLH14
Potri.003G135800	1.947721197	Up	6.16E-06	0.002308272	Steroid-transporting ATPase
Potri.001G024800	1.956803687	Up	1.75E-06	0.0009819	Annexin D3
Potri.003G068900	2.017352221	Up	3.58E-07	0.000283332	JAZ3
Potri.015G074600	2.054941717	Up	0.000370315	0.033472835	Polyamine oxidase
Potri.005G140700	2.055636521	Up	0.000115792	0.016419914	Floral homeotic protein APETALA 2
Potri.019G073400	2.119702922	Up	0.000358335	0.03278895	Metalloendoproteinase
Potri.004G222400	2.142395326	Up	9.38E-14	4.01E-10	ATP phosphoribosyltransferase
Potri.016G073400	2.196848775	Up	1.82E-07	0.000185074	Uncharacterized protein
Potri.001G378400	2.223752528	Up	1.09E-07	0.000122742	Gibberellin 2-beta-dioxygenase 1
Potri.008G184400	2.262785029	Up	9.62E-05	0.014485718	Cytochrome P450 71B10
Potri.011G096900	2.367809423	Up	1.30E-05	0.003835204	Hypothetical protein
Potri.009G145400	2.38825865	Up	6.50E-05	0.011119531	Cytochrome P450 94C1
Potri.019G031200	2.398482341	Up	9.40E-18	6.70E-14	Apyrase 2
Potri.016G137600	2.454196624	Up	4.35E-07	0.000321957	Costunolide synthase
Potri.001G024900	2.472406823	Up	9.52E-06	0.003130117	Annexin D4
Potri.017G013800	2.48692024	Up	2.30E-06	0.001199905	Tyrosine transaminase
Potri.016G117100	2.534609251	Up	2.90E-05	0.006532589	Leucocyanidin oxygenase
Potri.013G122400	2.563455246	Up	0.000443019	0.037577494	O-methyltransferase

Potri.001G388200	2.57203589	Up	1.09E-08	2.12E-05	Polyphenol oxidase
Potri.005G096266	2.790857365	Up	4.21E-05	0.008436991	Uncharacterized protein
Potri.002G065300	2.997732012	Up	1.38E-15	7.39E-12	Peroxidase 12
Potri.001G062500	3.278801885	Up	6.89E-13	2.46E-09	JAZ1
Potri.003G112400	3.367498423	Up	6.37E-07	0.000439095	Trehalose-phosphate phosphatase F
Potri.006G272700	3.759361998	Up	4.52E-08	7.35E-05	UDP-glucose 4,6-dehydratase
Potri.003G110901	4.618001199	Up	4.82E-08	7.35E-05	Autophagy-related protein 8F
Potri.003G170000	4.890409267	Up	1.33E-05	0.003835204	MADS box transcription factor

Table S5. Expression data for lignin synthesis genes in the *ap17ap45* mutants compared with wild-type plants.

Gene ID	Gene name	Fold Change	Log2 Fold Change	*p* value	padj	Up/Down	Significant
Potri.006G126800.v3.1	PAL1	0.695174026	-0.524553915	0.006304638	0.112428905	down	no
Potri.008G038200.v3.1	PAL2	0.672114397	-0.573221288	0.003318967	0.077158836	down	no
Potri.016G091100.v3.1	PAL3	0.730532857	-0.452978933	0.028300765	0.252312946	down	no
Potri.010G224100.v3.1	PAL4	0.665656389	-0.587150442	8.96E-06	0.0017663	down	no
Potri.010G224200.v3.1	PAL5	0.679099388	-0.558305363	5.50E-05	0.005648816	down	no
Potri.001G036900.v3.1	4CL3	0.766198295	-0.384210279	0.005476188	0.102570618	down	no
Potri.003G188500.v3.1	4CL5	0.70282697	-0.508758542	0.011409912	0.160626477	down	no
Potri.006G033300.v3.1	C3H3	0.599325701	-0.738587851	0.000822186	0.033366202	down	no
Potri.019G130700.v3.1	C4H2	0.625330677	-0.677308801	0.000316578	0.018129812	down	no
Potri.009G095800.v3.1	CAD1	0.629117671	-0.668598209	0.005372516	0.101886659	down	no
Potri.005G117500.v3.1	CAld5H1	0.609436803	-0.714451471	5.10E-05	0.00544695	down	no
Potri.007G016400.v3.1	CAld5H2	0.819922832	-0.28643996	0.114041621	0.465489008	down	no
Potri.009G099800.v3.1	CCoAOMT1	0.692532069	-0.530047215	0.029465712	0.256662797	down	no
Potri.001G304800.v3.1	CCoAOMT2	0.796208463	-0.32878189	0.153318259	0.522663456	down	no
Potri.008G136600.v3.1	CCoAOMT3	0.855800877	-0.224652937	0.316381678	0.693512722	down	no
Potri.003G181400.v3.1	CCR2	0.891824688	-0.165167958	0.179745573	0.559586306	down	no
Potri.012G006400.v3.1	COMT2	0.683665659	-0.548637135	0.003523829	0.079611083	down	no
Potri.003G059200.v3.1	CSE1	0.800839566	-0.320414841	0.043096098	0.305200842	down	no
Potri.001G175000.v3.1	CSE2	0.662829458	-0.593290373	0.00235048	0.064381281	down	no
Potri.003G183900.v3.1	HCT1	0.624239594	-0.679828228	0.000809668	0.032993957	down	no
Potri.001G042900.v3.1	HCT6	0.782252457	-0.354293809	0.313319147	0.691970123	down	no
